# ﻿On the species status of *Blattella germanica* and *Blattella asahinai* (Blattodea, Blattellidae), and other morphologically similar species

**DOI:** 10.3897/zookeys.1250.145981

**Published:** 2025-08-27

**Authors:** Jin-Zhuo Cai, Wen-Wen Yao, Zong-Qing Wang, Yan-Li Che

**Affiliations:** 1 College of Plant Protection, Southwest University, Beibei, Chongqing 400715, China Southwest University Beibei China; 2 Key Laboratory of Agricultural Biosafety and Green Production of Upper Yangtze River (Ministry of Education), Southwest University, Chongqing 400715, China Southwest University Beibei China

**Keywords:** ABGD, Blattodea, *Blattella germanica asahinai*, *
Jacobsonina
*, species delimitation, subspecies

## Abstract

*Blattella germanica* (Linnaeus, 1767), a member of the Blattellidae family within the order Blattodea, is a significant global sanitary pest. Several species within the genus *Blattella* Caudell, 1903 and its closely related genera (*Episymploce* Bey-Bienko, 1950; *Symploce* Hebard, 1916; and *Jacobsonina* Hebard, 1929) exhibit external morphological traits similar to those of *B. germanica*. By integrating morphological identification and molecular analyses, one new species was identified: *Jacobsonina uncata* Cai, Yao & Che, **sp. nov.** Additionally, *Blattella asahinai* Mizukubo, 1981 was downgraded to a subspecies of *B. germanica*. The application of molecular data, specifically cytochrome oxidase c subunit I (COI), has proven to be a straightforward and effective method for distinguishing *B. germanica* from its morphologically similar relatives.

## ﻿Introduction

Due to its rapid reproduction rate, strong adaptability, and effective concealment, *Blattella germanica* is widely distributed worldwide (Ren et al. 2023). It serves as a vector for various bacteria, viruses, and parasites that can transmit pathogens to humans through contact with contaminated food surfaces, ultimately leading to food poisoning and other health complications (Akinjogunla et al. 2012; Moges et al. 2016; Naqqash et al. 2014). Additionally, allergens present in their molts and excrement can trigger allergic reactions and asthma attacks in sensitive individuals (Gore and Schal 2007; Santos et al. 1999).

Several species within the *Blattella* genus, such as *Blattella asahinai* Mizukubo, 1981, *Blattella lituricolis* (Walker, 1868), and *Blattella nipponica* Asahina, 1963, as well as species from closely related genera like *Jacobsonina aliena* (Brunner von Wattenwyl, 1893), exhibit a high degree of morphological similarity to *B. germanica*. Males of these morphologically similar species are slender, measuring approximately 15.0 mm in body length, and displaying coloration that ranges from pale to dark yellowish brown, with or without markings on the pronotum. They possess well-developed eyes, distinct ocelli, specialized terga, and a supra-anal plate. The significant morphological similarity between *B. germanica* and its morphologically similar species poses a challenge for accurate differentiation based on subtle variations in male characteristics, such as the pronotum, specialized dorsal glands, and external genitalia (Roth 1985, 1986; Wang et al. 2010), and this may lead to potential errors in population estimates and miscalculations of the amount of control chemicals needed for effective pest management. Therefore, there is an urgent need for a systematic comparison of these subtle variations to clarify the distinguishing characteristics of these morphologically similar species and to develop novel methods for rapidly differentiating *B. germanica* from its closely related species.

In recent years, COI-based DNA barcoding (658 bp) has emerged as the most widely utilized molecular method for distinguishing cockroach species, demonstrating high precision (Deng et al. 2020; Wang et al. 2021; Han et al. 2022; Li et al. 2023). Favorable results have also been reported for the genera *Blattella* and *Jacobsonina* (Yao et al. 2024). Among these species, *B. asahinai* closely resembles *B. germanica* morphologically and displays a smaller COI-based genetic distance (0.92%), whereas the genetic divergences between *B. germanica* and other *Blattella* species range from 9.44% to 20.25% (Yao et al. 2024). Moreover, *B. asahinai* is inferred to have diverged from *B. germanica* approximately 2,100 years ago (Tang et al. 2024). Thus, the taxonomic status of *B. germanica* and *B. asahinai* requires clarification through further research. Additionally, the external female genitalia have proven useful in identifying certain cockroach species (*Cryptocercus*: Bai et al. 2018; *Anaplecta*: Zhu et al. 2022; *Eupolyphaga*: Han et al. 2024). However, it remains uncertain whether the external female genitalia can be used to distinguish *B. germanica* from morphologically similar species.

Consequently, this study integrates morphological and molecular data to accurately distinguish *B. germanica* from its morphologically similar species. This establishes reliable criteria for their identification and explores the role of female external genitalia in the classification of *Blattella* species. These findings provide a foundation for the precise identification and evolutionary analysis of the genus *Blattella* and its close relatives.

## ﻿Materials and methods

Samples were collected from the southeastern and southwestern regions of China. Specimens were preserved in analytical-grade ethanol and stored at -80 °C until processing. All voucher and type specimens (with details provided in Table 1) examined in this study were deposited at the College of Plant Protection, Southwest University, Chongqing, China (**SWU**).

**Table 1.** Samples used in molecular species delimitation.

Species	Location	Voucher number	GenBank ID
*B. bisignata*	Huizhou, Guangdong	BB1	PP730854
	Nanning, Guangxi	BB2	PP730855
	Panzhihua, Sichuan	BB3	PP730856
	Baoting, Hainan	BB4	PP730857
	Guigang, Guangxi	/	KY349784.1
	Guangzhou, Guangdong	BB5	PP730858
*B. germanica*	laboratory culture	BG1	PP730863
	Zhongshan, Guangdong	/	KF640071.1
*B. lituricolis*	Zhongshan, Guangdong	/	KX962535
	Pu’er, Yunnan	BL1	PP730864
	Yinggeling, Hainan	BL2	PP730865
	Ganzhou, Jiangxi	BL3	PP730866
*B. nipponica*	Chiba, Japan	/	LC619061
	Fuzhou, Fujian	BN1	PP730868
	Guiyang, Guizhou	BN2	PP730869
	Hangzhou, Zhejiang	BN3	PP730870
	Nanjing, Jiangsu	BN4	PP730871
	Huangshan, Anhui	BN5	PP730872
	Huanggang, Hubei	BN6	PP730873
	Qianjiang, Chongqing	BN7	PP730874
	Meishan, Sichuan	BN8	PP730875
	Weihai, Shandong	BN9	PP730876
	Shaoyang, Hunan	BN10	PP730877
	Chongzuo, Guangxi	BN11	PP730878
	Huanggang, Hubei	BN12	PP730879
*B. germanica asahinai*	State of Florida, United States of America	/	MG458949
	Dehong, Yunnan	BA3	PP730853
*B. sauteri*	Putian, Fujian	/	KY349679
	Putian, Fujian	BS1	PP730881
	Putian, Fujian	BS3	PP730883
	Bangzhou, Hunan	BS2	PP730882
*B. radicifera*	Xishuangbanna, Yunnan	/	KY349677.1
	Xishuangbanna, Yunnan	/	KY349676.1
	Xishuangbanna, Yunnan	BR1	PP730880
*B. ligulata*	Xishuangbanna, Yunnan	BLI1	PP730867
	Dianyuan,Yunnan	BlatLigu	PV939656
*B. foliolata*	Panzhihua, Sichuan	BF1	PP730862
*B. biligata*	Yuxi, Yunnan	BBL1	PP730859
	Yuxi, Yunnan	BBL2	PP730860
*B. confusa*	Yingjiang, Yunnan	BC1	PP730861
	Tengchong, Yunnan	BlatConf	PV939657
*B. punctoria*	Xishuangbanna, Yunnan	BlatPunc1	PP730894
	Xishuangbanna, Yunnan	BlatPunc2	PV939658
	Xishuangbanna, Yunnan	BlatPunc3	PV939659
*B. subvittata*	Sanya, Hainan	BlatBisp2	PV939676
	Sanya, Hainan	BlatBisp1	PV939675
*J. aliena*	Pu’er, Yunnan	JA1	PP730884
	Qianxinan, Guizhou	JA2	PP730885
	Pingtangxian, Guizhou	JacoAlie	OQ736932
*J. platysoma*	Yuxi, Yunnan	JP1	PP730886
*J. tortuosa*	Jinghong, Yunnan	JT1	PP730887
*J. plicata*	Xishuangbanna, Yunnan	JacoPlic1	PP730888
	Xishuangbanna, Yunnan	JacoPlic2	PP730889
*J. subapiculata*	Yingjiang, Yunnan	JacoSuba1	PP730890
	Yingjiang, Yunnan	JacoSuba2	PP730891
	Yingjiang, Yunnan	JacoSuba3	PP730892
*J. ericonvexa*	Ruili, Yunnan	JacoEric	PP730893
*J. uncata* sp. nov.	Xishuangbanna, Yunnan	JacoUnca	PV939674
*E. longiloba*	Mt. Dawei, Yunnan	EpisLong	PV939660
*E. wulingensis*	Mt. Limu, Hainan	EpisWuli	PV939661
	Mt. Limu, Hainan	EpisWuli2	OQ736929
*E. torchaceus*	Baoting, Hainan	EpisTorc1	PV939662
	Baoting, Hainan	EpisTorc2	PV939663
	Maoganxiang, Hainan	EpisTorc3	OQ736927
*E. brevis*	Honghe, Yunnan	EpisBrev1	PV939664
	Qiannan, Guizhou	EpisBrev2	PV939665
*E. kryzhanovshii*	Guiyang, Guizhou	EpisKryz1	PV939666
	Xinping, Yunnan	EpisKryz2	PV939667
	Xinping, Yunnan	EpisKryz3	PV939668
*E. spinosa*	Mt. Dawei, Yunnan	EpisSpin	PV939669
*E. paradoxura*	Mt. Dawei, Yunnan	EpisPara1	PV939670
	Mt. Dawei, Yunnan	EpisPara2	PV939671
	Mt. Dawei, Yunnan	EpisPara3	PV939672
*S. marginata*	Mt. Jinyun, Chongqing	SympMarg1	PV944124
	Mt. Jinyun, Chongqing	SympMarg2	PV939673
*M. concava*	/	/	MF136390
*D. punctata*	/	/	MF479156

Morphological terminology used mainly follows Roth (2003) for external morphology, McKittrick (1964) for female external genitalia, Klass (1997) for male external genitalia, and Li et al. (2018) for venation. The abbreviations for female genitalia in this study are as follows: **aa.**-anterior arch; **bsv.**-basivalvula; **cp.**-crosspiece; **intc.s.**-intercalary sclerite; **p.l.**-posterior lobes of valvifer II; **pp.**-paraprocts; **pt.**-paratergites; **SVII**-sternum VII; **T I–X**-abdominal tergites I–X; **v.I, II, III**- valvules I–III; **vlf.I**-first valvifer; **vlf.Ia**-first valvifer arm; **vst.s.**-vestibular sclerite; and **sp.pl.**-spermathecal plate. The abbreviations for venation are: **CuA**-cubitus anterior; **CuP**-cubitus posterior; **M**-media; **Pcu**-postcubitus; **R**-radius; **RA**-radius anterior; **RP**-radius posterior; **ScP**-subcosta posterior; and **V**-vannal.

Genital segments of the examined specimens were placed in centrifuge tubes and soaked in 10% NaOH. The tubes were then immersed in hot water (~90 °C) for 15–20 minutes to remove excess fat. Afterward, the genital segments were rinsed with distilled water, and observed in glycerin using a Motic K400 stereomicroscope. Photographs of the specimens and genitalia were captured with a Leica M205A stereomicroscope. All images were edited and assembled into plates using Adobe Photoshop CC 2019.

DNA extraction, PCR, and sequencing were conducted following the methodology outlined by Yao et al. (2024). All 22 newly acquired sequences were submitted to GenBank (https://www.ncbi.nlm.nih.gov/nuccore) with accession numbers PV939656 to PV939676, and PV944124 (Table 1).

A total of 77 COI sequences (658 bp) were analyzed, including 46 sequences from *Blattella*, 29 from its closely related genera (*Episymploce*, *Symploce* and *Jacobsonina*) (Wang et al. 2023), and two outgroups (*Diploptera punctata* (Eschscholtz, 1822) and *Margattea concava* Wang, Che & Wang, 2009). These sequences were aligned using MEGA 11.0 (Kumar et al. 2007) and visually adjusted after translation into amino acid sequences. Genetic divergence values, both intraspecific and interspecific, were quantified using the Kimura 2-parameter (K2P) distance model (Kimura 1980) in MEGA 11.0. The maximum likelihood (ML) tree was constructed in PhyloSuite v. 1.2.3 (Zhang et al. 2020) using IQ-TREE v. 2.1.3 (Nguyen et al. 2014) with models (COI_pos 1, TRN+I+G; COI_pos 2, TIM+I; COI_pos 3, GTR+G) selected by PartitionFinder v. 2.1.1 (Lanfear et al. 2017) based on the corrected Akaike Information Criterion (AICc). Ten independent likelihood searches (each for 10,000 ultrafast bootstrap replicates) were performed, and the result with the highest likelihood was selected. The molecular species delimitation was conducted using the Automatic Barcode Gap Discovery (ABGD) method (Puillandre et al. 2012) on the online platform (https://bioinfo.mnhn.fr/abi/public/abgd/). Default parameters were used, except for the relative gap width, which was set to 1.0, and the Jukes-Cantor (JC69) model was applied.

## ﻿Results

### ﻿Morphology

From the general appearance perspective, it is quite challenging to distinguish *B. germanica* and its morphologically similar species. However, by integrating external morphological traits with genital characteristics – i.e. body size, body coloration, head, male pronotum, specialized abdominal terga, dorsal glands, supra-anal plate, subgenital plate, phallomere, and sclerite structures of female genitalia – we identified a total of 28 morphospecies, which includes one new species, *J. uncata* Cai, Yao & Che, sp. nov. The identified morphospecies are distributed as follows: *Episymploce* (7 morphospecies), *Symploce* (1 morphospecies), *Jacobsonina* (7 morphospecies), and *Blattella* (13 morphospecies) among 75 specimens. The results of the morphological comparisons are presented below.

**Figure 1.**
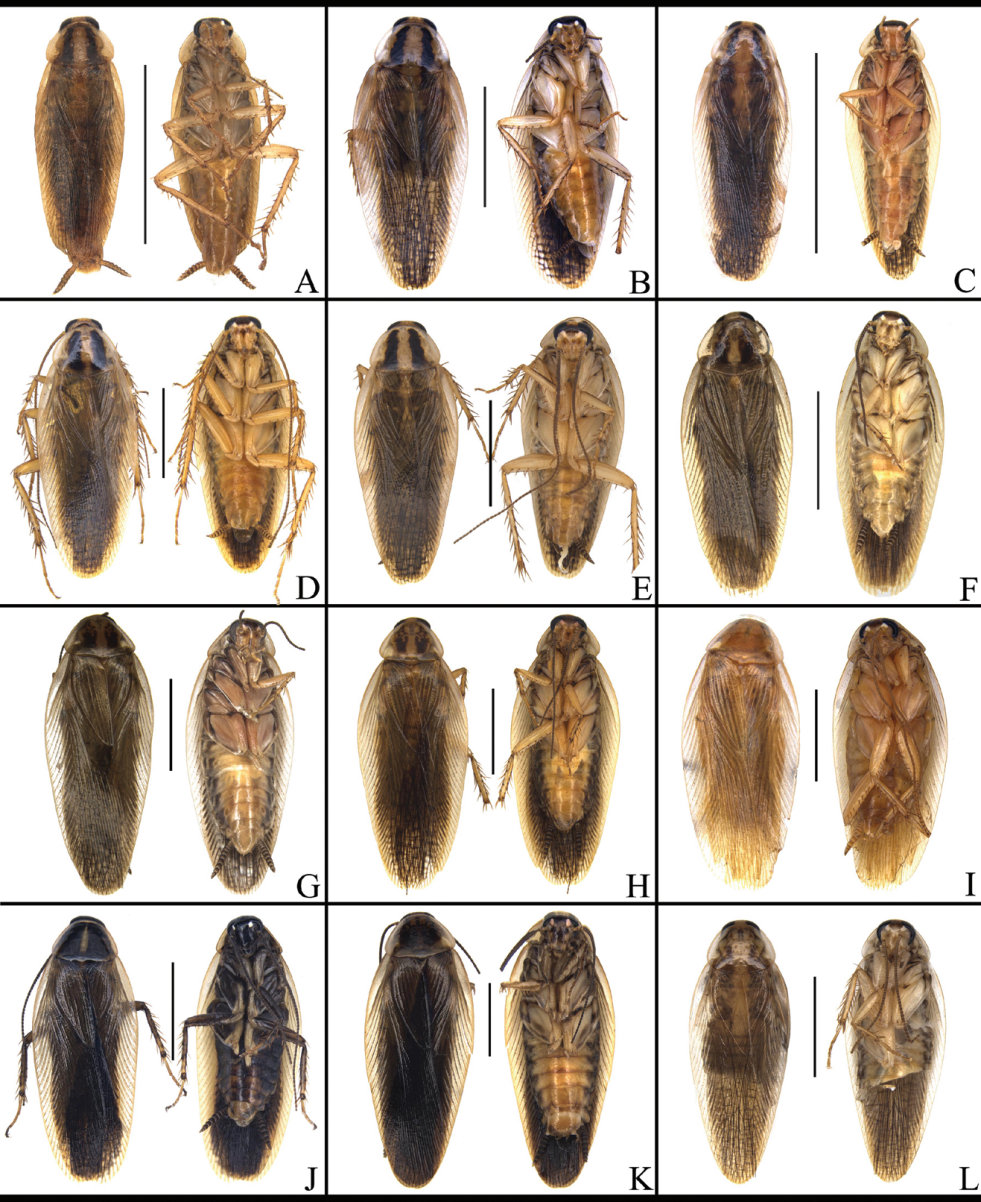
Male habitus of *B. germanica* and of its similar species. A. *B. germanica*; B. *B. lituricolis*; C. *B. asahinai*; D. *B. nipponica*; E. *B. bisignata*; F. *B. sauteri*; G. *B. radicifera*; H. *B. punctoria*; I. *B. biligata*; J. *B. confusa*; K. *B. ligulata*; L. *B. subvittata* Scale bars: 5 mm.

**Figure 2.**
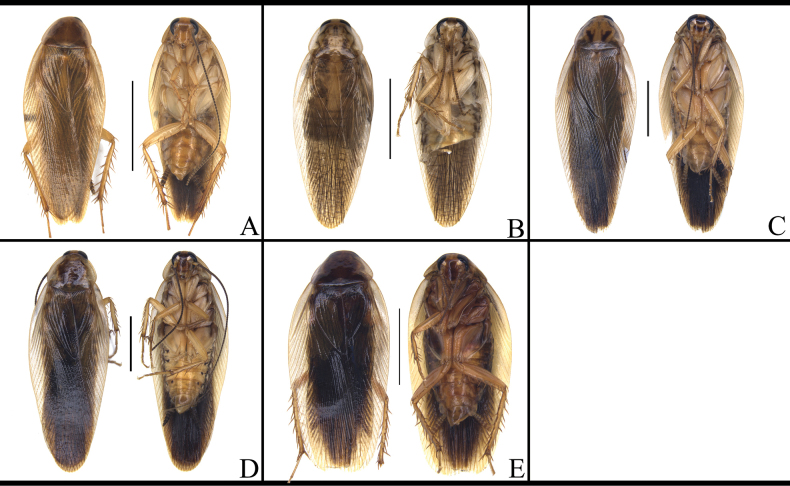
Male habitus of *B. germanica* and of its similar species. A. *J. aliena*; B. *J. uncata* sp. nov.; C. *E. torchaceus*; D. *E. spinosa*; E. *S. marginata*. Scale bars: 5 mm.

Compared to most species, *Blattella ligulata* (Bey-Bienko, 1957), *Episymploce torchaceus* (Feng & Woo, 1999), and *Episymploce spinosa* (Bey-Bienko, 1969) demonstrate significantly larger body sizes (Figs 1K, 2C, D), while *J. aliena* and *J. uncata* sp. nov. are generally smaller (Fig. 2A, B). In terms of body coloration, most species are pale yellowish brown to yellowish brown, while *B. ligulata*, *B. confusa*, *E. torchaceus*, *E. spinosa*, and *Symploce marginata* Bey-Bienko, 1927 are overall dark brown (Figs 1J, K, 2C–E).

**Figure 3.**
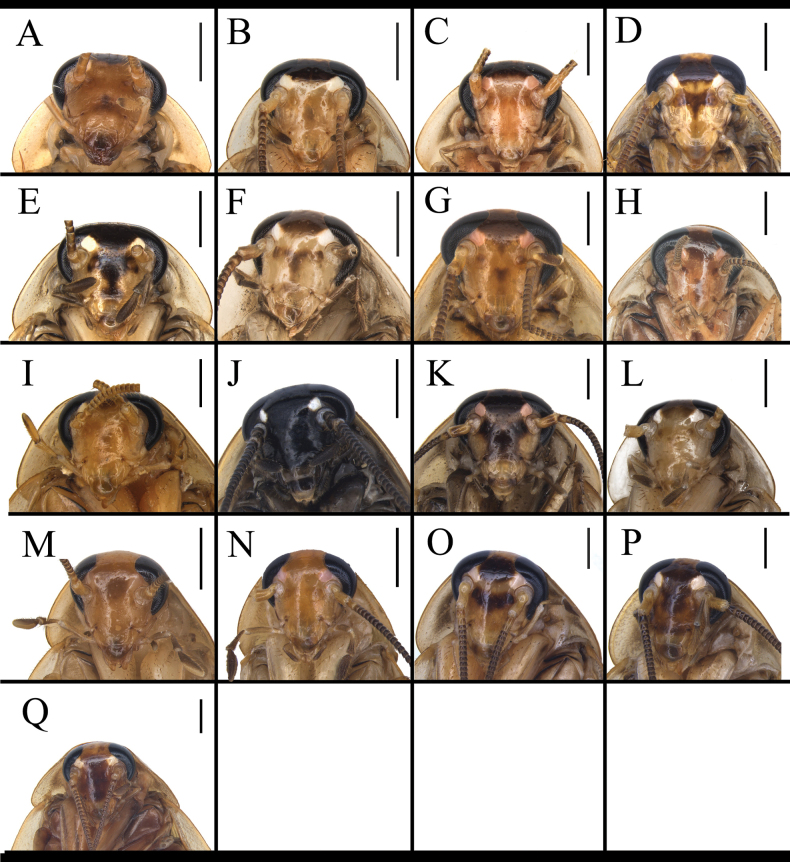
Head of *B. germanica* and of its similar species. A. *B. germanica*; B. *B. lituricolis*; C. *B. asahinai*; D. *B. nipponica*; E. *B. bisignata*; F. *B. sauteri*; G. *B. radicifera*; H. *B. punctoria*; I. *B. biligata*; J. *B. confusa*; K. *B. ligulata*; L. *B. subvittata*; M. *J. aliena*; N. *J. uncata* sp. nov.; O. *E. torchaceus*; P. *E. spinosa*; Q. *S. marginata*. Scale bars: 1 mm.

Most of *B. germanica* and its similar species are hard to distinguish by characteristics of male head, except for *B. confusa*, which has a black head (Fig. 3J). In addition, the faces of *B. germanica*, *B. lituricolis*, *B. asahinai*, *B. radicifera*, *Blattella punctoria* Yao & Che, 2024, *Blattella biligata* (Walker, 1868), *B. subvittata*, and *J. aliena* are without distinct markings (Fig. 3A–C, G–I, L, N), while other species have distinct markings (Fig. 3D–F, K, O–Q).

**Figure 4.**
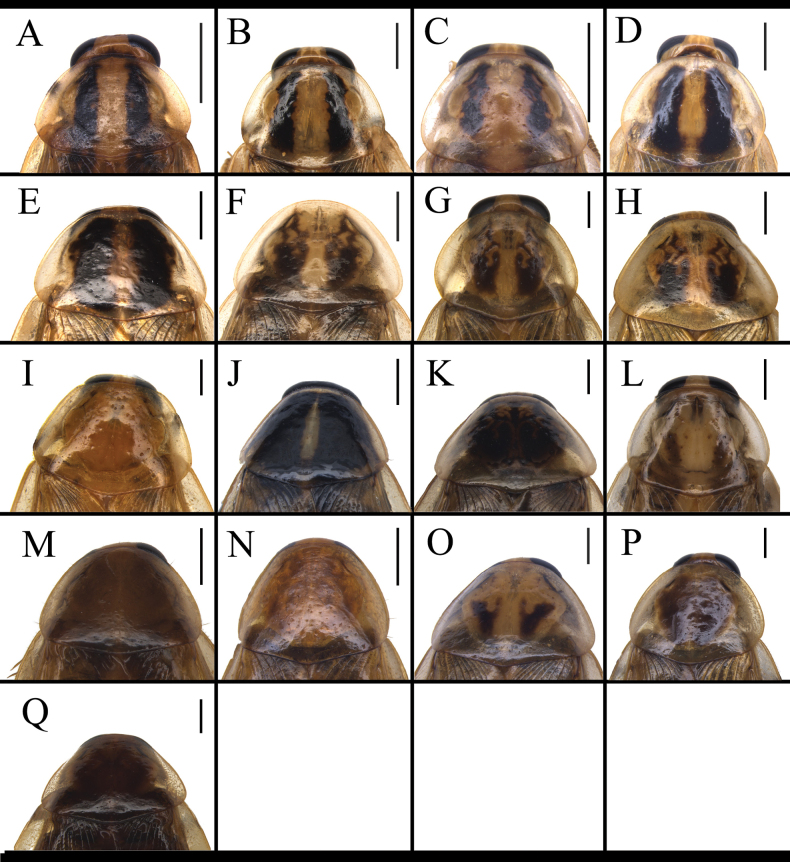
Male pronotum of *B. germanica* and of its similar species. A. *B. germanica*; B. *B. lituricolis*; C. *B. asahinai*; D. *B. nipponica*; E. *B. bisignata*; F. *B. sauteri*; G. *B. radicifera*; H. *B. punctoria*; I. *B. biligata*; J. *B. confusa*; K. *B. ligulata*; L. *B. subvittata*; M. *J. aliena*; N. *J. uncata* sp. nov.; O. *E. torchaceus*; P. *E. spinosa*; Q. *S. marginata*. Scale bars: 1 mm.

Markings of male pronotum are similar in *B. germanica*, *B. lituricolis*, *B. asahinai*, *B. bisignata*, and *B. nipponica*, all with two black longitudinal strips (Fig. 4A–E). The pronotum of *B. sauteri*, *B. radicifera*, *B. punctoria*, *B. ligulata*, *E. torchaceus*, *E. spinosa*, and *S. marginata* also have distinct markings (Fig. 4F–H, K, O–Q). the pronotum of *B. confusa* has two large black markings and a thin white marking in the middle part, which is easily recognizable (Fig. 4J) while the pronota of *B. biligata* and *J. aliena* lack distinct markings (Fig. 4I, M).

**Figure 5.**
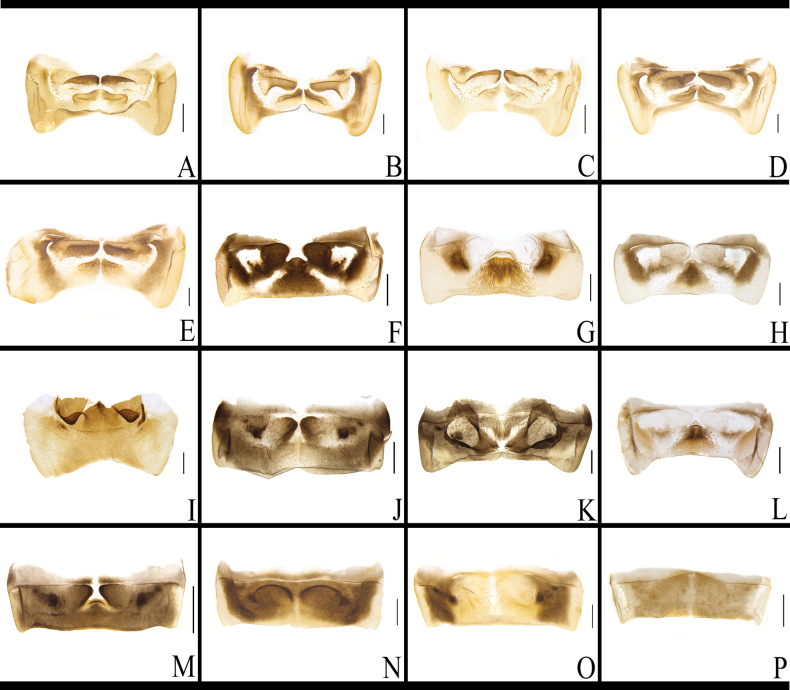
Abdominal tergite 7 of *B. germanica* and of its similar species. A. *B. germanica*; B. *B. lituricolis*; C. *B. asahinai*; D. *B. nipponica*; E. *B. bisignata*; F. *B. sauteri*; G. *B. radicifera*; H. *B. punctoria*; I. *B. biligata*; J. *B. confusa*; K. *B. ligulata*; L. *B. subvittata*; M. *E. spinosa*; N. *S. marginata*; O. *E. torchaceus*; P. *J. aliena*. Scale bars: 0.5 mm.

**Figure 6.**
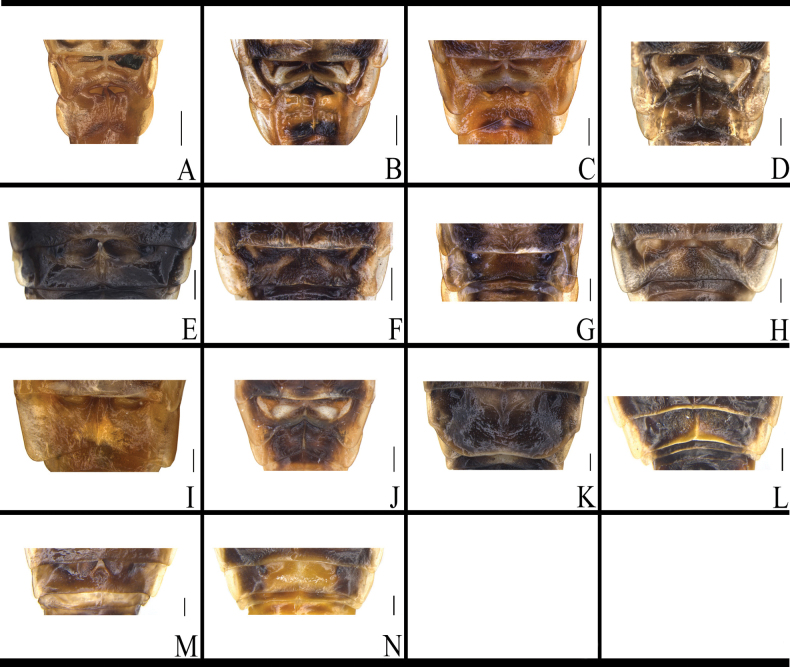
Dorsal glands of *B. germanica* and of its similar species. A. *B. germanica*; B. *B. lituricolis*; C. *B. asahinai*; D. *B. bisignata*; E. *Blattella confusa*; F. *B. sauteri*; G. *B. radicifera*; H. *B. punctoria*; I. *B. biligata*; J. *B. nipponica*; K. *B. ligulata*; L. *S. marginata*; M. *E. spinosa*; N. *E. torchaceus*. Scale bars: 0.5 mm.

The abdominal tergites, TVII and TVIII are specialized in *B. germanica*, *B. lituricolis*, *B. asahinai*, *B. bisignata* and *B. nipponica*. TI and TVII are specialized in *S. marginata*. *B. sauteri*, *B. punctoria*, *B. radicifera*, *B. biligata*, *B. confusa*, *B. ligulata*, *B. subvittata*, and *E. torchaceus* all have only one specialized abdominal tergite, TVIII. Specialization of TI, VII, and IX occurs in *E. spinosa*. All species of *Jacobsonina* lack specialized abdominal tergites. TVII of *B. germanica*, *B. lituricolis*, and *B. asahinai* has two transverse grooves near the anterior margin of the middle area (Fig. 5A–C, Table 2). TVII of *B. germanica* and *B. lituricolis* have two transverse grooves on the posterior margin of the median of TVII, but the grooves of *B. lituricolis* are narrower. *B. asahinai* lacks grooves on the posterior margin of TVII. A pair of open grooves is present in the anterior margin of the median domain of TVII in *B. bisignata* and *B. nipponica*. In addition, *B. bisignata* has a narrow groove in the posterior margin of TVII, but *B. nipponica* does not (Fig. 5D, E, Table 2). The groove on TVII of *B. bisignata* is not covered by TVI and was obviously exposed, the groove on TVII of *B. nipponica* is not significantly exposed (Fig. 6D, E, Table 2).

**Table 2.** Comparison of important distinguishing features of *B. germanica* and its morphologically similar relatives.

Species	Abdominal tergite 7	Glandular fossae of abdominal tergite 8	Abdominal tergite 1	Abdominal tergite 9	Supra-anal plate	Subgenital plate
*B. germanica*	two transverse grooves near the anterior margin of the middle area	exposed and broad, the posterior margin setose	/	/	left and right paraprocts broad and similar, both bifurcated	with a flat apical part and a subrectangular notch at the left corner
*B. lituricolis*	the grooves narrower	distinct, glandular openings not attached	/	/	left and right paraprocts broad and similar, both bifurcated	with a flat apical part and a subrectangular notch at the left corner
*B. asahinai*	no grooves on the posterior margin	partially obscured by tergite 7 and only two upward-elevated hairy margins visible	/	/	left and right paraprocts broad and similar, both bifurcated	with a flat apical part and a subrectangular notch at the left corner
*B. nipponica*	the groove not significantly exposed	inconspicuously exposed	/	/	left and right paraprocts broad and similar, both bifurcated	with a flat apical part and a subrectangular notch at the left corner
*B. bisignata*	the groove not covered by tergite 6 and obviously exposed	obviously exposed	/	/	left and right paraprocts broad and similar, both bifurcated	with a flat apical part and a subrectangular notch at the left corner
*B. sauteri*	the triangular region of posterior margin with setae	/	/	/	the base of the right paraproct expanded and tapered toward the end	with small spines on the middle of both sides
*B. radicifera*	the posterior margin of the ridge with a densely setose area	/	/	/	right paraproct with a trifurcate protrusion	with small spines on the middle of both sides
*B. punctoria*	the triangular region of posterior margin with setae	/	/	/	with a small spine at each posterior lateral corner	with a small spine on the left side
*B. biligata*	a small area between the two fossae bearing dense setae	/	/	/	with a broad and rounded posterior margin	no spines on either margin
*B. confusa*	a central ridge and a deep rounded fossa on each side of the ridge	/	/	/	right paraproct tapers to a point	with a small protuberance between the styli
*B. ligulata*	a triangular and setose area with two large fossae on either side	/	/	/	right paraproct slender, arched and curved	outer margin of the left stylus spiny
*B. subvittata*	two distinct fossae and a setose area	/	/	/	the middle part of the posterior margin bearing two spines	protuberance in the middle lateral margins long
*J. aliena*	unspecialized	/	/	/	right paraproct pincer-like	lower margin truncate
*J. uncata* sp. nov.	unspecialized	/	/	/	left posterior lateral corner bearing three spinules and right bearing two spinules	left hind lateral corner bearing many spinules
*E. torchaceus*	sparsely covered with setae	/	/	/	left margin bearing three spinules and right margin bearing four spinules	left stylus folded from center to the upper left
*E. spinosa*	an obscure protrusion with a small dimple on both sides	/	specialized with the middle part bearing tufted setae	with a serrated and slightly curved margin	left posterior margin bearing two spine-like protrusions	left margin arched and outer margin densely covered with small spines
*S. marginata*	two fossae with small setae	/	specialized with the middle part bearing tufted setae	/	with a small protrusion on its left lateral margin	left stylus points from left towards right with the apical part sharp

There is no obvious difference between the TVII of *B. sauteri* and *B. punctoria*. The anterior margin of the median domain of TVII has an upwardly protruding ridge, the triangular region of posterior margin has setae, and a rounded socket is present on each side (right and left), which is covered by TVI when not dissected (Fig. 6F, H, Table 2). The remaining species have clearly differentiated characteristics of TVII, making it easy to distinguish. That of *B. radicifera* has a sub-trapezoidal elevated ridge in the middle part, the posterior margin of the ridge has a densely setose area, and a rounded fovea is present on each side of the ridge (Fig. 5G, Table 2). TVII of *B. biligata* is distinctly broad, with the anterior half having a pair of deep fossae, a longitudinal ridge between the two fossae, and a small area between the two fossae bearing dense setae (Fig. 6I, Table 2). TVII of *B. confusa* is distinctly broad, with a central ridge and a deeper rounded fossa on each side of the ridge (Fig. 5J, Table 2). The middle part of TVII of *B. ligulata* has a triangular and setose area, with two large fossae on either side (Fig. 5K, Table 2). TVII of *B. subvittata* has two distinct fossae and a setose area (Fig. 5L, Table 2). The middle part of TVII of *E. spinosa* has an obscure protrusion, with a small dimple on both sides (Figs 5M, 6M, Table 2). TVII of *S. marginata* has two fossae, and the anterior margin of the fossae has small setae (Figs 5N, 6L, Table 2). The middle part of TVII of *E. torchaceus* is sparsely covered with setae (Figs 5O, 6N, Table 2). *Jacobsonina aliena* does not have a specialized abdominal TVII (Fig. 5P, Table 2).

**Figure 7.**
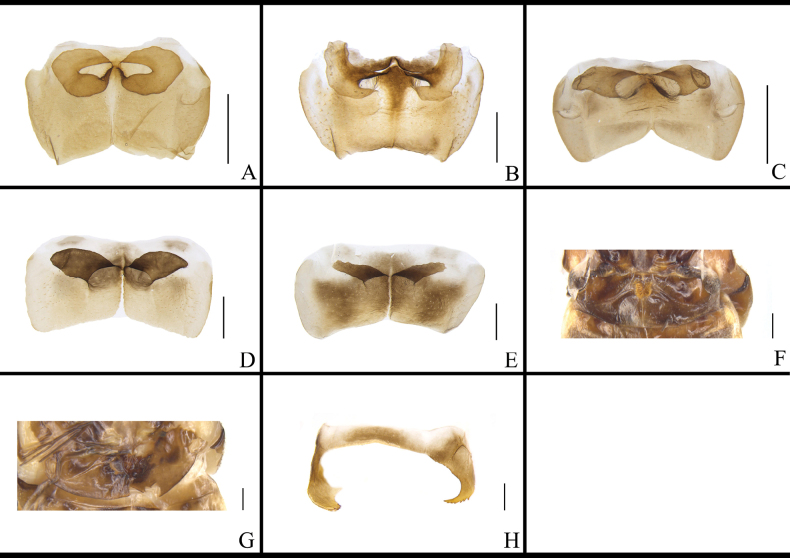
Abdominal tergites 1, 8 and 9 of *B. germanica* and of its similar species. A. *B. germanica*; B. *B. lituricolis*; C. *B. asahinai*; D. *B. nipponica*; E. *B. bisignata*; F. *S. marginata*; G. *E. spinosa*; H. *E. spinosa*. Scale bars: 0.5 mm.

Morphological differences of TVIII among *B. germanica*, *B. lituricolis*, *B. asahinai*, *B. bisignata*, and *B. nipponica* are pronounced. The median longitudinal groove of tergite 8 in *B. germanica* is conspicuous, dorsal glandular fossae exposed, broad, and suborbicular or ellipsoid. The glandular openings are connected or nearly connected at the anterior margin, and the posterior margin is setose (Fig. 7A, Table 2). The glandular fossa in TVIII of *B. lituricolis* is covered by TVII near the anterior margin (Fig. 6B, Table 2). The glandular fossa is distinct, with glandular openings not attached; the inner margin extends toward the center, and the basal extension is thicker than the terminal one (Fig. 7B, Table 2). Glandular fossae in TVIII of *B. asahinai* are partially obscured by TVII, only with two upward-elevated hairy margins visible (Fig. 6C, Table 2). Two dorsal glandular fossae are oblate, their anterior margins nearly connected, posterior margins medially setose, unconnected, and slightly upwardly curved (Fig. 7C, Table 2).

**Figure 8.**
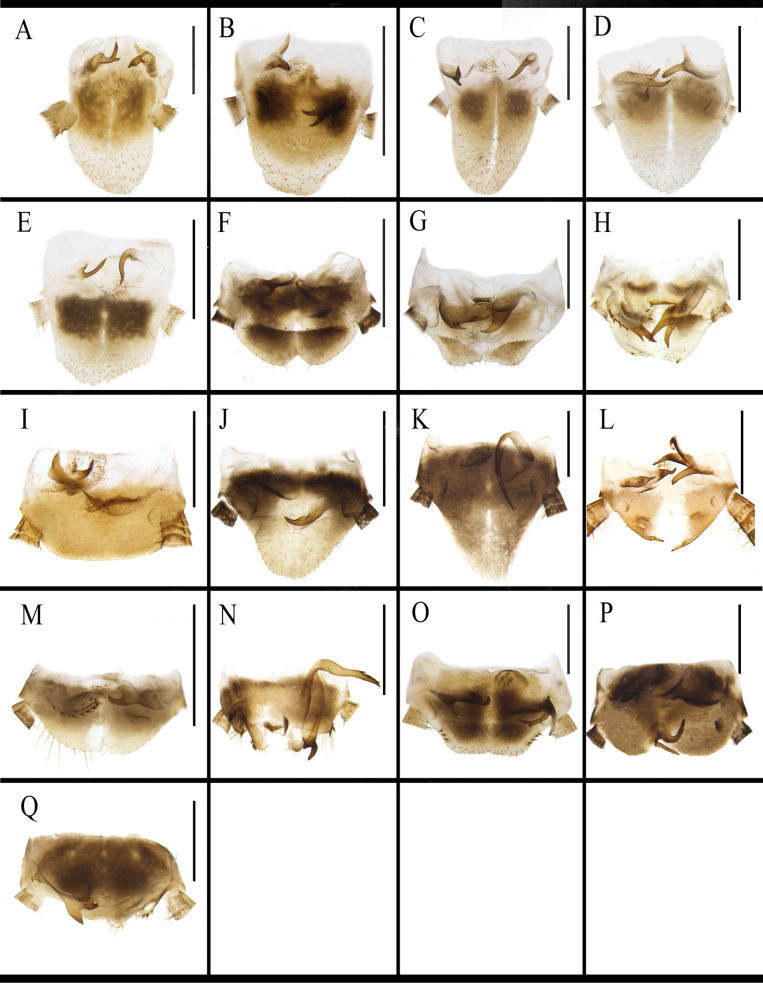
Supra-anal plate of *B. germanica* and of its similar species. A. *B. germanica*; B. *B. lituricolis*; C. *B. asahinai*; D. *B. nipponica*; E. *B. bisignata*; F. *B. sauteri*; G. *B. radicifera*; H. *B. punctoria*; I. *B. biligata*; J. *B. confusa*; K. *B. ligulata*; L. *B. subvittata*; M. *J. aliena*; N. *J. uncata* sp. nov.; O. *E. torchaceus*; P. *E. spinosa*; Q. *S. marginata*. Scale bars: 1 mm.

When not dissected, the glandular fossa in TVIII of *B. bisignata* is obviously exposed (Fig. 6D, Table 2), whereas that of *B. nipponica* is inconspicuously exposed (Fig. 6E, Table 2). TVIII of *B. bisignata* and *B. nipponica* has a longitudinal ridge in the middle, two flaky glandular fossae, a nearly connected anterior margin of the fossae, and posterior margins that are not closed and slightly retracted toward the middle ridge (Fig. 7D, E, Table 2).

TI of *S. marginata* and *E. spinosa* is specialized, with the middle part bearing tufted setae (Fig. 7F, G, Table 2). The ventral margin of the right dorsal lobe of TIX in *E. spinosa* extends backward, with a serrated and slightly curved margin (Fig. 7H, Table 2).

The supra-anal plates of *B. germanica*, *B. lituricolis*, *B. asahinai*, *B. nipponica*, and *B. bisignata* are similar in morphology: distinctly longer than the subgenital plates overall; left and right paraprocts broad and similar, both being bifurcated (Fig. 8A–E, Table 2). The posterior margin of supra-anal plate of *B. sauteri* is concave inward at the middle part; the apex of the left paraproct is bifurcated, and the base of the right paraproct is expanded and tapered toward the end (Fig. 8F, Table 2). The posterior margin of supra-anal plate of *B. radicifera* is concave inward at the middle part, with a small spinule at each posterior lateral angle. The left paraproct has a bifurcate protrusion, and the right paraproct has a trifurcate protrusion (Fig. 8G, Table 2).

The supra-anal plate of *B. punctoria* has a small spine at each posterior lateral corner; both paraprocts are bifurcate. The left paraproct bears multiple small spines at basal part, and the right paraproct has two small spines in middle of both branches (Fig. 8H, Table 2). The supra-anal plate of *B. biligata* is symmetrical, with a broad and rounded posterior margin. Both paraprocts are pointed at apex, with the left paraproct positioned above and the right paraproct below (Fig. 8I, Table 2). The supra-anal plate of *B. confusa* is ligulate, with the posterior margin slightly smooth in middle part. The left paraproct gradually expands from the base toward the tip, and the right paraproct gradually tapers to a point (Fig. 8J, Table 2). The supra-anal plate of *B. ligulata* is ligulate. The left paraproct is broad at basal part, with apical part bearing four small bifurcations. The right paraproct is slender, arched (Fig. 8K, Table 2). The supra-anal plate of *B. subvittata* is nearly symmetrical, with the middle part containing a hyaline area and the middle part of the posterior margin bearing two spines. The left paraproct has a terminal curved hook-like protrusion and a tapering protrusion; the right paraproct has a protrusion slightly twisted at the apex (Fig. 8L, Table 2). The supra-anal plate of *J. aliena* is symmetrical, with a smooth posterior margin. The left paraproct is finger-like, with the lower margin densely covered with small spines; the right paraproct is pincer-like (Fig. 8M, Table 2). The supra-anal plate of *J. uncata* sp. nov. is unsymmetrical, with the left posterior lateral corner bearing three spinules and the right bearing two spinules. The left paraproct is simple, with the apical part bearing four spinules and the basal part having one curved and short fork (the fork’s apex with 5 spinules). The right paraproct is complex: the apical part without spinules, one of its two forks is robust with a strongly curved hooked end, and the other fork is long and bent towards the right (Fig. 8N, Table 2). The supra-anal plate of *E. torchaceus* is symmetrical and subtrapezoidal, with the middle of posterior margin slightly concave inwards, the left margin bearing three spinules, and the right margin bearing four spinules. The left paraproct is bifurcated, with one long and one short bifurcation. The right paraproct has a rounded area bearing spinules and a trifurcated protrusion (Fig. 8O, Table 2). The supra-anal plate of *E. spinosa* is asymmetrical, with the left posterior margin bearing two spine-like protrusions (1 straight, the other curved upwards). The left paraproct has two small forks, the right paraproct has one small protrusion (Fig. 8P, Table 2). The posterior margin of supra-anal plate of *S. marginata* is slightly concave inwards medially. The apical part of the left paraproct is bifurcated: one surface bears spinules, and the other has an acutely protruding apex. The supra-anal plate exhibits a small protrusion on its left lateral margin (Fig. 8Q, Table 2).

**Figure 9.**
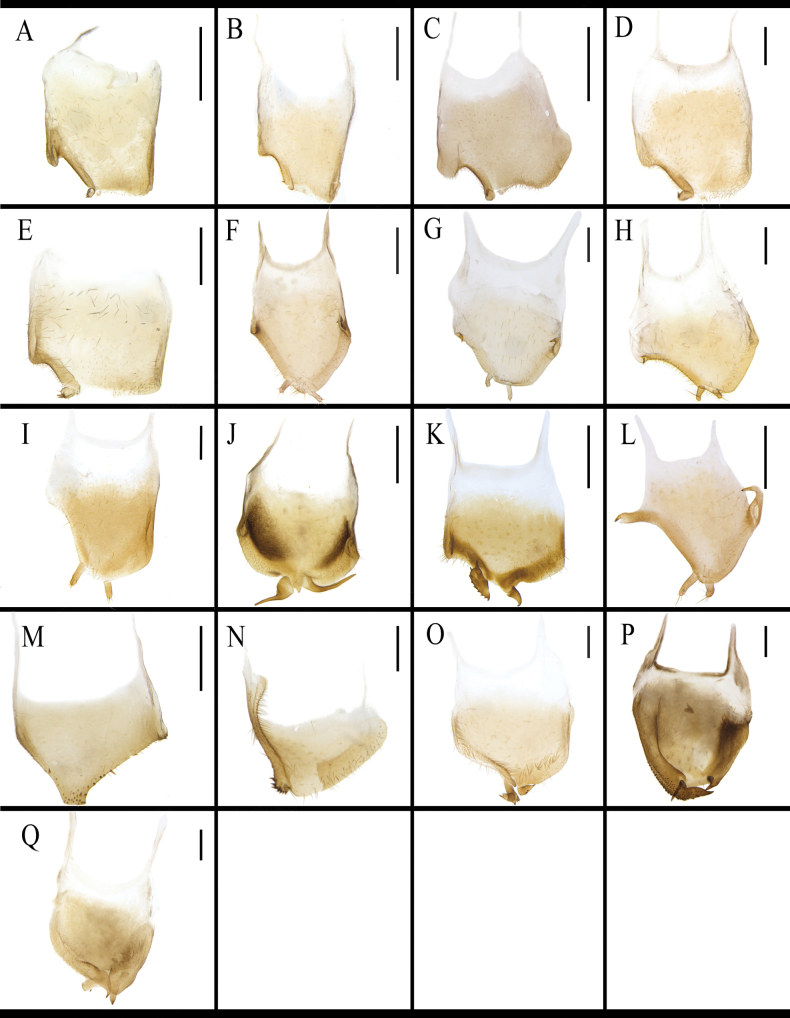
Subgenital plate of *B. germanica* and of its similar species. A. *B. germanica*; B. *B. lituricolis*; C. *B. asahinai*; D. *B. nipponica*; E. *B. bisignata*; F. *B. sauteri*; G. *B. radicifera*; H. *B. punctoria*; I. *B. biligata*; J. *B. confusa*; K. *B. ligulata*; L. *B. subvittata*; M. *J. aliena*; N. *J. uncata* sp. nov.; O. *E. torchaceus*; P. *E. spinosa*; Q. *S. marginata*. Scale bars: 1 mm.

The subgenital plates of *B. germanica*, *B. lituricolis*, *B. asahinai*, *B. nipponica*, and *B. bisignata* are similar: featuring a flat apical part and a subrectangular notch at the left corner (Fig. 9A–E, Table 2). The left stylus is situated at the end of the notch, with the apex bearing several small spinules. The right stylus is small, rounded, and is positioned very close to the left stylus.

The subgenital plates of *B. sauteri*, *B. radicifera*, *B. punctoria* and *B. biligata* are similar and asymmetrical, with posterior margins protruding medially and two finger-like styli (Fig. 9F–I, Table 2). Both *B. sauteri* and *B. radicifera* bear small spines on the middle of both sides of the subgenital plate, but *B. radicifera* is broader overall than *B. sauteri*. The right stylus is located on the right side of the posterior margin in *B. sauteri*, while in *B. radicifera*, it is located at the middle of the posterior margin (Fig. 9F, G, Table 2). The subgenital plate of *B. biligata* lacks spines on either margin. *B. punctoria* bears a small spine on the left side only, with the notch in the left posterior lateral corner being more pronounced (Fig. 9H, I, Table 2).

The subgenital plates of *B. confusa*, *B. ligulata*, and *B. subvittata* are readily distinguishable. The two styli of *B. confusa* are long, with the basal part expanded, the apical part pointed, and a small protuberance between the styli (Fig. 9J, Table 2). The outer margin of the left stylus of *B. ligulata* is spiny (Fig. 9K, Table 2). The protuberance in the middle lateral margins of *B. subvittata* is long (Fig. 9L, Table 2).

The subgenital plate of *J. aliena* is asymmetrical, with the posterior margin medially protruded and the lower margin truncate. Margins bear some small spines. Styli absent (Fig. 9M, Table 2). The subgenital plate of *J. uncata* sp. nov. is asymmetrical, with the left side thickened and the left hind lateral corner bearing many spinules. Styli absent (Fig. 9N, Table 2). The subgenital plate of *E. torchaceus* is asymmetrical, with the posterior margin slightly protruded medially, the right margin rounded, and the left margin slightly concave internally in the terminal half. Two styli are inserted at the middle of the posterior margin. The left stylus is folded from center to the upper left, with small spines at the folded corners. The right stylus is smaller and bears three small spines (Fig. 9O, Table 2).

The subgenital plate of *E. spinosa* is asymmetrical, with the left margin arched, the outer margin densely covered with small spines, and the apical extension acuminate. The spiny tip of the right extension is distinctly smaller than left. The left stylus is long; The right stylus is short (Fig. 9P, Table 2). The subgenital plate of *S. marginata* is asymmetrical, with the posterior margin bearing two projections: one longer, projecting from the right side towards the left, and the other stout, short and projecting downwards. The left stylus points from the left towards the right, with the apical part sharp; the right stylus extends vertically downwards (Fig. 9Q, Table 2).

**Figure 10.**
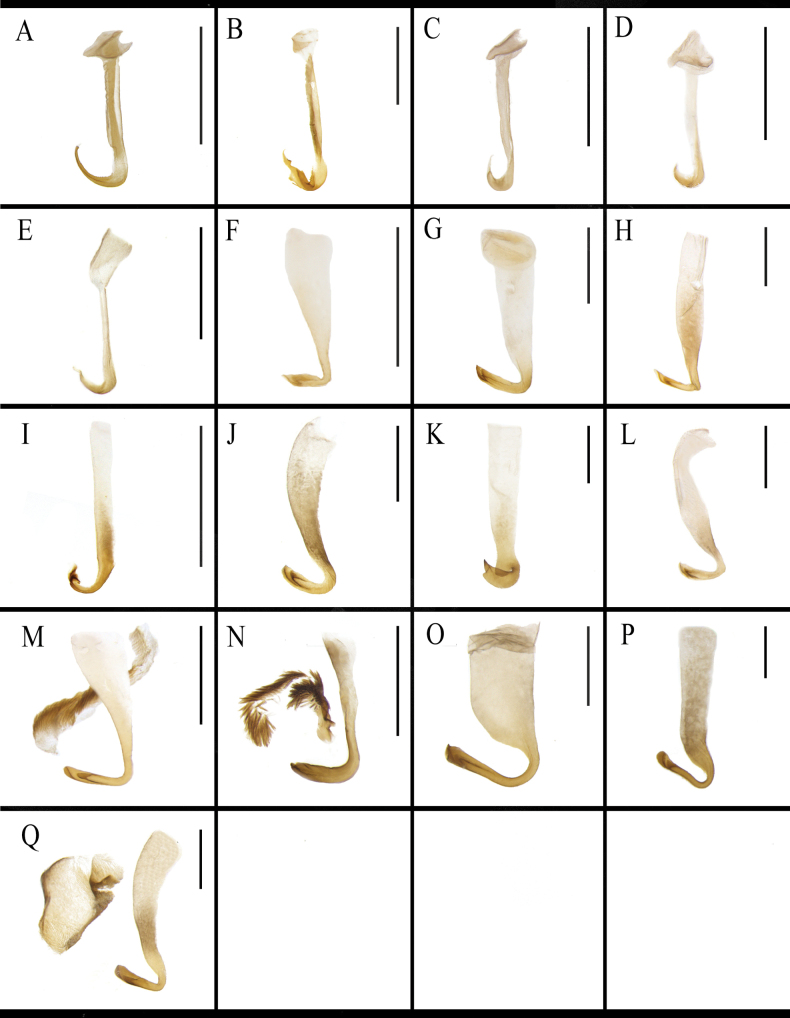
Left phallomere of *B. germanica* and of its similar species. A. *B. germanica*; B. *B. lituricolis*; C. *B. asahinai*; D. *B. nipponica*; E. *B. bisignata*; F. *B. sauteri*; G. *B. radicifera*; H. *B. punctoria*; I. *B. biligata*; J. *B. confusa*; K. *B. ligulata*; L. *B. subvittata*; M. *J. aliena*; N. *J. uncata* sp. nov.; O. *E. torchaceus*; P. *E. spinosa*; Q. *S. marginata*. Scale bars: 1 mm.

**Figure 11.**
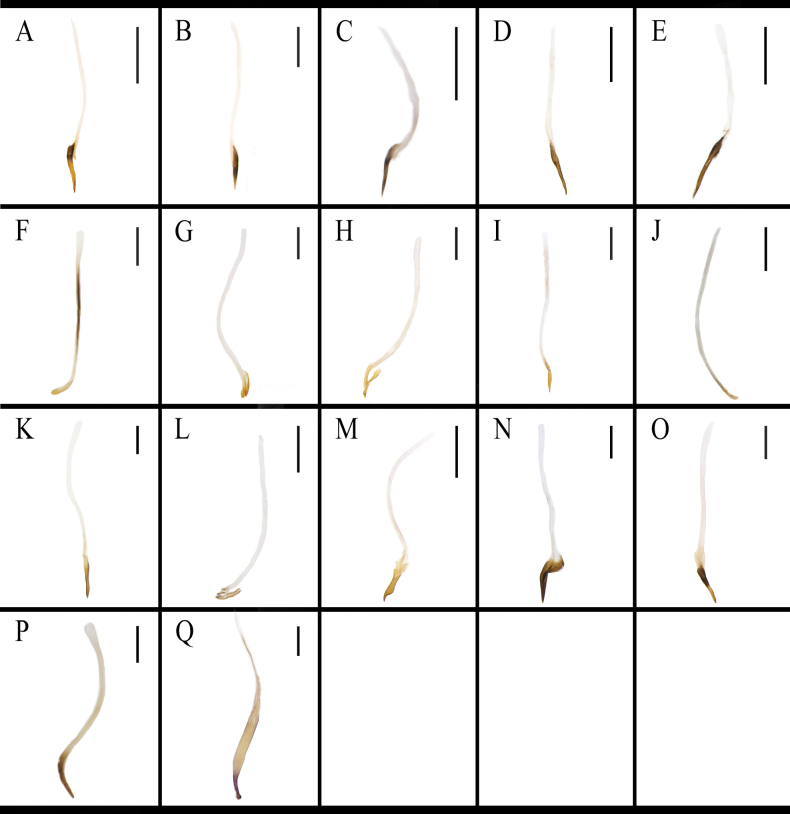
Middle phallomere of *B. germanica* and of its similar species. A. *B. germanica*; B. *B. lituricolis*; C. *B. asahinai*; D. *B. nipponica*; E. *B. bisignata*; F. *B. sauteri*; G. *B. radicifera*; H. *B. punctoria*; I. *B. biligata*; J. *B. confusa*; K. *B. ligulata*; L. *B. subvittata*; M. *J. aliena*; N. *J. uncata* sp. nov.; O. *E. torchaceus*; P. *E. spinosa*; Q. *S. marginata*. Scale bars: 1 mm.

**Figure 12.**
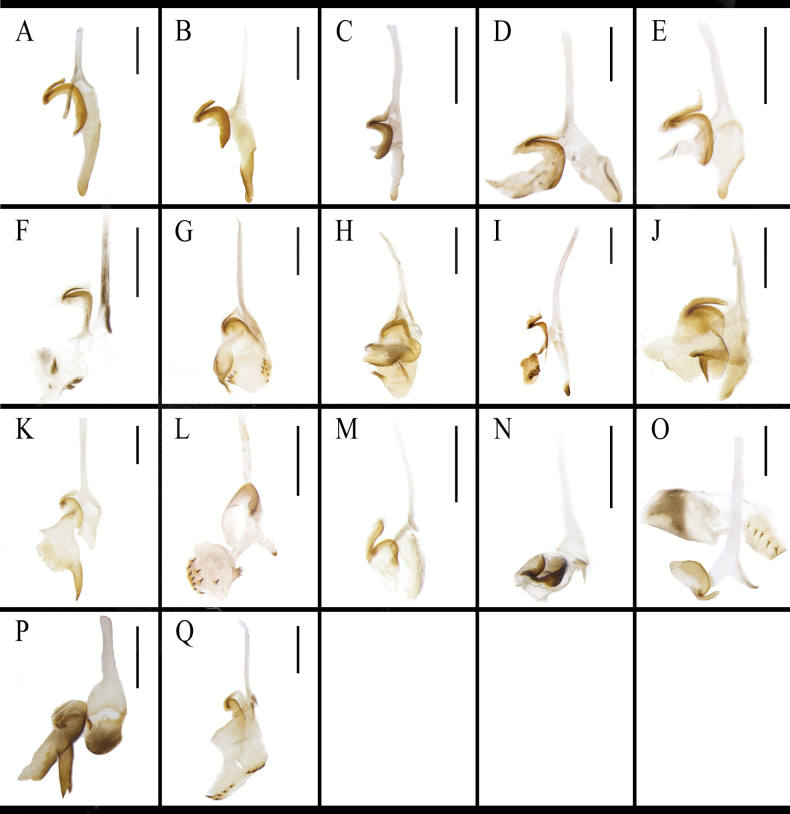
Right phallomere of *B. germanica* and of its similar species. A. *B. germanica*; B. *B. lituricolis*; C. *B. asahinai*; D. *B. nipponica*; E. *B. bisignata*; F. *B. sauteri*; G. *B. radicifera*; H. *B. punctoria*; I. *B. biligata*; J. *B. confusa*; K. *B. ligulata*; L. *B. subvittata*; M. *J. aliena*; N. *J. uncata* sp. nov.; O. *E. torchaceus*; P. *E. spinosa*; Q. *S. marginata*. Scale bars: 1 mm.

The morphology of the left phallomere is similar in most *B. germanica* and its morphologically similar species, but there are some exceptions. The left phallomere of *B. germanica*, *B. lituricolis*, *B. asahinai*, *B. nipponica*, and *B. bisignata* is similar, while the inner edge of the curved hook of *B. lituricolis* is dentate and the others are smooth (Fig. 10A–E). The curved hook portion of the left phallomere of *B. ligulata* is short, broad, and twisted with a sharp apex (Fig. 10K). The left phallomere of *J. aliena* has a banded and hairy accessory sclerite (Fig. 10M). The left phallomere of *J. uncata* sp. nov. bears two bristly accessory sclerites (Fig. 10N). The left phallomere of *E. torchaceus* is unusually robust except for the curved hook-like portion (Fig. 10O). The accessory sclerite of *S. marginata* is lamellar, broad (Fig. 10Q). The morphology of the middle phallomere is similar in most *B. germanica* and its morphologically similar species, all possessing an elongate, poorly sclerotized basal part and an acute, strongly sclerotized apical part. The apical part of the middle phallomere of *B. punctoria* has a bifurcation (Fig. 11H); the apical part of *B. subvittata* bears an elongate bony sclerite (Fig. 11L); and the middle part of *S. marginata* is wide (Fig. 11Q). The right phallomere of *B. germanica* and its morphologically similar species has an inverted Y-shaped elongate sclerite and a curved lamellate sclerite. In addition, the right phallomere of many species also has other sclerites. *Blattella sauteri* has a spiny, irregular sclerite on the surface (Fig. 12F); *B. radicifera* has an oval sclerite bearing some spines at the lateral margins, with a basal expanded protrusion attached to the left side (Fig. 12G); *B. punctoria* has an oval sclerite bearing three long spines at the basal part, to which also attached a lamellate, bristly-margined sclerite (Fig. 12H); *B. biligata* has a lamellar and spiny sclerite (Fig. 12I); *B. confusa* has a bifurcated sclerite (Fig. 12J); *B. ligulata* has a large, spiky-ended sclerite (Fig. 12K); *B. subvittata* has a sclerite with ten small spines on the margin and a short protruding, the apex of which with four spines (Fig. 12L). *Jacobsonina aliena* has a thin, flaky, poorly sclerotized sclerite (Fig. 12M). *Episymploce torchaceus* has a broad sclerite with five small spines on the right middle part (Fig. 12O); *E. spinosa* has a bifurcate sclerite and an ovoid and lamellar sclerite (Fig. 12P). *Symploce marginata* has a sclerite bearing small spines on posterior margin (Fig. 12Q).

**Figure 13.**
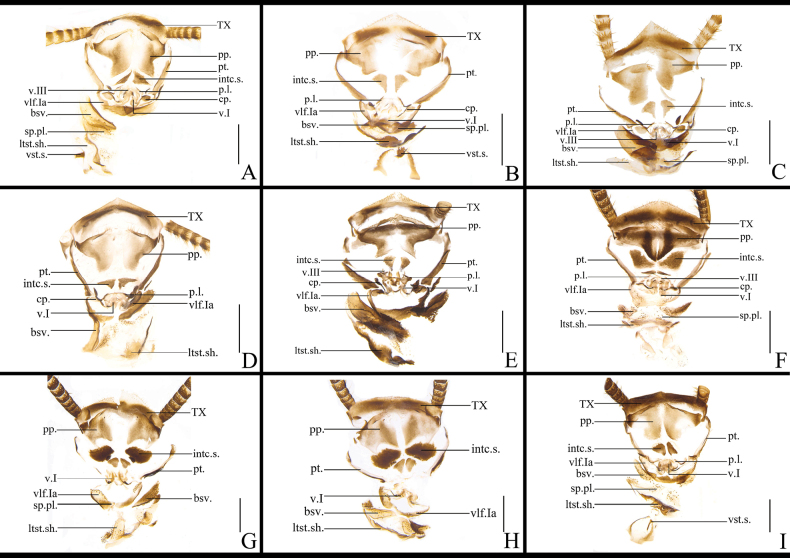
Female external genitalia of *B. germanica* and of its similar species. A. *B. germanica*; B. *B. lituricolis*; C. *B. asahinai*; D. *B. bisignata*; E. *B. nipponica*; F. *B. sauteri*; G. *B. radicifera*; H. *B. punctoria*; I. *B. ligulata*. Scale bars: 1 mm. Abbreviations: aa.-anterior arch; bsv.-basivalvula; cp.-crosspiece; intc.s.-intercalary sclerite; p.l.-posterior lobes of valvifer II; pp.-paraprocts; pt.-paratergites; SVII-sternum VII; T I–X-abdominal tergites I–X; v.I, II, III- valvules I–III; vlf.I-first valvifer; vlf.Ia-first valvifer arm; vst.s.-vestibular sclerite; and sp.pl.-spermathecal plate.

**Figure 14.**
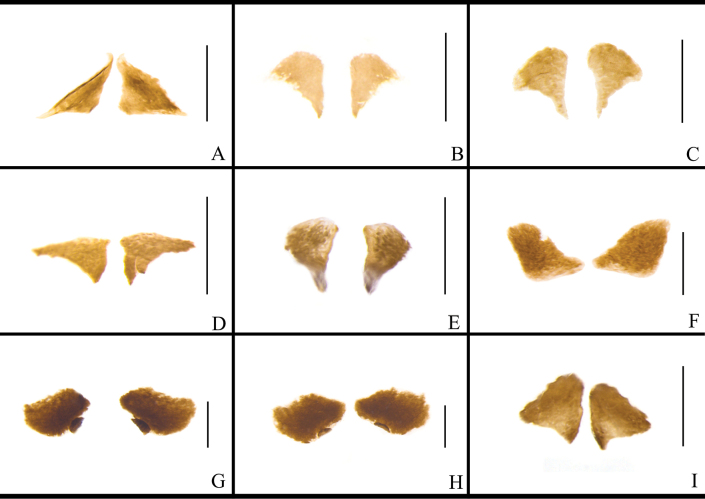
The intc.s. of *B. germanica* and of its similar species. A. *B. germanica*; B. *B. lituricolis*; C. *B. asahinai*; D. *B. bisignata*; E. *B. nipponica*; F. *B. sauteri*; G. *B. radicifera*; H. *B. punctoria*; I. *B. ligulata*. Scale bars: 0.5 mm.

**Figure 15.**
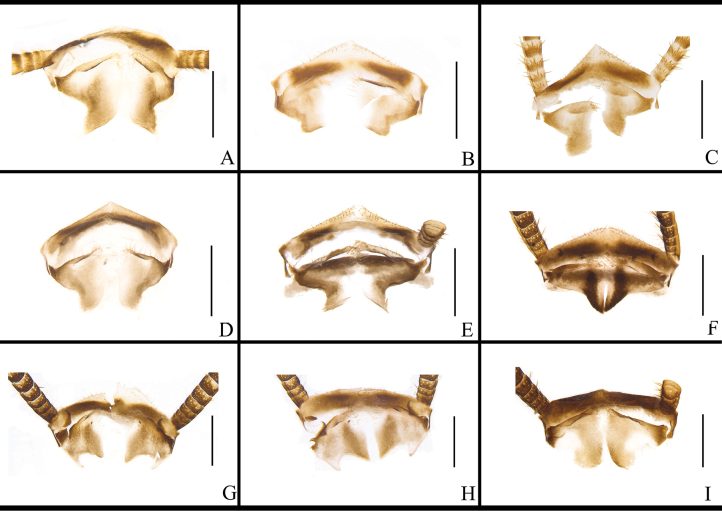
The pp. of *B. germanica* and of its similar species. A. *B. germanica*; B. *B. lituricolis*; C. *B. asahinai*; D. *B. bisignata*; E. *B. nipponica*; F. *B. sauteri*; G. *B. radicifera*; H. *B. punctoria*; I. *B. ligulata*. Scale bars: 0.5 mm.

The structure of the female external genitalia of *Blattella* species is broadly similar (Fig. 13). The posterior margin of supra-anal plate prominent. Paraprocts are broadly lamellar. Paratergites are banded. Intercalary sclerite are symmetrical, lamellar, mostly subtriangular, and slightly rounded in a few cases. Valves are not well sclerotized, short. Crosspieces are distinct, with a slender basal part and an oval or irregular shaped apical part. Posterior lobes of valvifer II are sub-rectangular. First valvifer arm are nearly symmetrical, almost U-shaped, relatively flat, and with a spiny or smooth surface. Basivalvula are lamellar and slightly broad, with a surface that may or may not bear spines. Laterosternal shelf are well sclerotized or not, irregular shaped. Vestibular sclerite are well sclerotized and either lamellar or long-banded. Among these species, the main sclerite structures in which differences exist are the following: the intc.s. of *B. germanica* and *B. bisignata* are nearly right triangles (Fig. 14A, D); those of *B. lituricolis*, *B. asahinai*, and *B. nipponica* are fan-shaped (Fig. 14B, C, E); intc.s. of *B. radicifera* and *B. punctoria* are similar with rounded edges and an overall approximate trapezoid shape (Fig. 14G, H); and the intc.s. of *B. ligulata* is nearly heart-shaped (Fig. 14I). The middle part of the outer margins of the pp. of *B. germanica*, *B. lituricolis*, *B. asahinai*, *B. bisignata*, *B. nipponica*, and *B. ligulata* is concave inwardly (Fig. 15A–E, I); *B. germanica* and *B. nipponica* both with a sharp apex (Fig. 15A, E), while *B. lituricolis*, *B. asahinai*, *B. bisignata*, and *B. ligulata* have a more rounded apex (Fig. 15D, I). The pp. of *B. sauteri* is the most recognizable, with its posterior margins sharp and converging toward the middle (Fig. 15F) while the pp. of *B. radicifera* and *B. punctoria* are similar (Fig. 15G, H).

**Figure 16.**
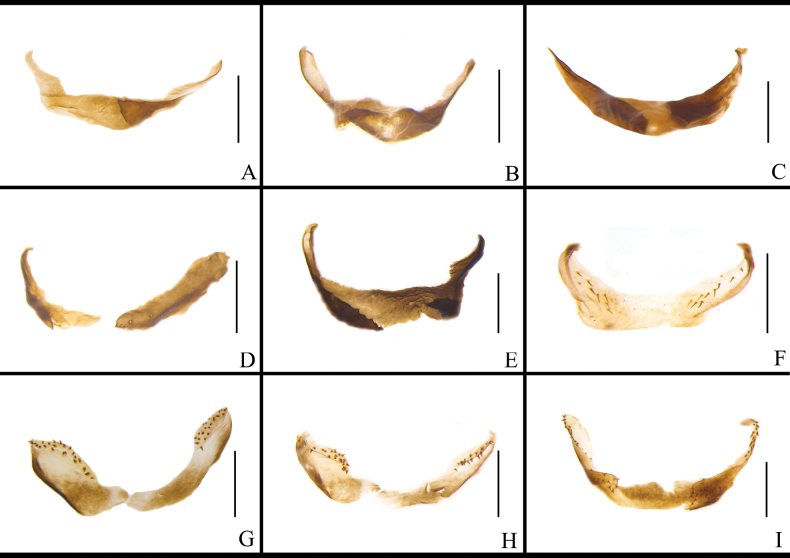
The vlf.Ia of *B. germanica* and of its similar species. A. *B. germanica*; B. *B. lituricolis*; C. *B. asahinai*; D. *B. bisignata*; E. *B. nipponica*; F. *B. sauteri*; G. *B. radicifera*; H. *B. punctoria*; I. *B. ligulata*. Scale bars: 0.5 mm.

The vlf.Ia of *B. germanica*, *B. lituricolis*, *B. asahinai*, *B. bisignata*, and *B. nipponica* is not setose (Fig. 16A–E), but that of *B. sauteri*, *B. radicifera*, *B. punctoria*, and *B. ligulata* is setose (Fig. 16F–I). The setae are evenly distributed on the surface of vlf.Ia in *B. sauteri* (Fig. 16F), but are only distributed on the edges of the vlf.Ia in *B. radicifera*, *B. punctoria*, and *B. ligulata* (Fig. 16G–I).

### ﻿Molecular analysis based on COI sequences

**Figure 17.**
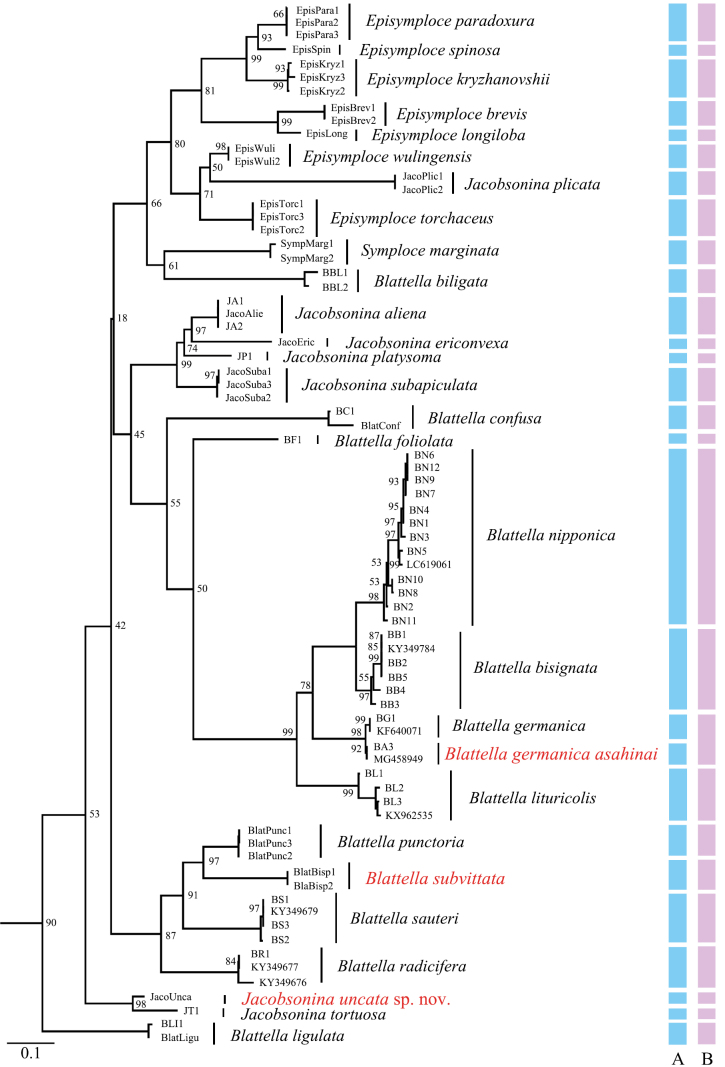
Maximum likelihood (ML) tree based on COI sequences. Branch labels represent bootstrap support percentages. Colored bars indicate species delimitation results obtained through different methods. A. Morphology (blue); B. ABGD results (purple).

The maximum likelihood (ML) tree constructed from COI sequences demonstrated that samples identified as the same morphospecies based on morphological traits were clearly grouped together. Additionally, most terminal branches exhibited relatively strong support values (Fig. 17). The ABGD analysis categorized 75 ingroup COI sequences into 27 molecular operational taxonomic units (MOTUs), which closely aligned with the results of the morphological delimitation. The only discrepancy was the relationship between *B. germanica* and *B. asahinai*.

**Figure 18.**
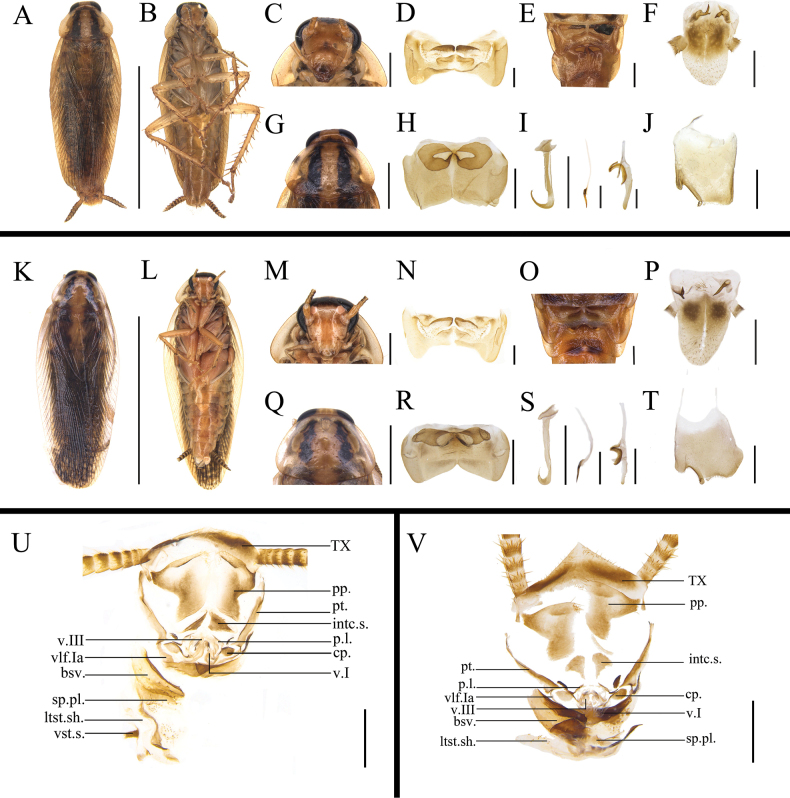
Comparative morphology of *B. germanica* and *B. asahinai*. A–J, U. *B. germanica*; K–T, V. *B. asahinai*. A. Habitus, dorsal view; B. Habitus, ventral view; C. Head, ventral view; D. Abdominal tergite 7, ventral view; E. Dorsal glands, dorsal view; F. Supra-anal plate, ventral view; G. Pronotum, dorsal view; H. Abdominal tergite 8, ventral view; I. Phallomere; J. Subgenital plate, ventral view; K. Habitus, dorsal view; L. Habitus, ventral view; M. Head, ventral view; N. Abdominal tergite 7, ventral view; O. Dorsal glands, dorsal view; P. Supra-anal plate, ventral view; Q. Pronotum, dorsal view; R. Abdominal tergite 8, ventral view; S. Phallomere; T. Subgenital plate, ventral view; U, V. Female external genitalia. Scale bars: 5 mm (A, B, K, L); 1 mm (C, F, G, I, J, M, P, Q, S–V); 0.5 mm (D, E, H, N, O, R). Abbreviations: aa.-anterior arch; bsv.-basivalvula; cp.-crosspiece; intc.s.-intercalary sclerite; p.l.-posterior lobes of valvifer II; pp.-paraprocts; pt.-paratergites; SVII-sternum VII; T I–X-abdominal tergites I–X; v.I, II, III- valvules I–III; vlf.I-first valvifer; vlf.Ia-first valvifer arm; vst.s.-vestibular sclerite; and sp.pl.-spermathecal plate.

The morphological similarities between *B. germanica* and *B. asahinai* are evident in several aspects: body coloration yellowish brown (Fig. 18A, B, K, L), interocular space approximately equal to the distance between ocelli (Fig. 18C, M), male pronotum with two black longitudinal strips (Fig. 18G, Q), the male abdominal tergites 7 and 8 specialized (Fig. 18D, H, N, R), left and right paraprocts of supra-anal plate bifurcated (Fig. 18F, P), and subgenital plates with a sub-rectangular notch at the left corner (Fig. 18J, T). The sclerite structures of female external genitalia also show striking resemblances, including TX, pp., pt., intc.s., cp., p.l., vlf.Ia, bsv., and ltst.sh (Fig. 18U, V). Despite these similarities, several differences were observed: (1) the male abdominal tergite 8: the posterior margin of the glandular fossae in *B. asahinai* not curved forward and not extending beyond the anterior margin, in contrast to *B. germanica* (Fig. 18H, R) (Roth 1986); (2) the first ootheca (refers to the first ootheca cockroach produced, the original author’s meaning) and first instars of *B. asahinai* smaller than those of *B. germanica*; with colorless (white) spots present at the margins and central region of abdominal tergite in late instars of *B. asahinai*, whereas these spots on *B. germanica* are lightly pigmented (*n* = 10 specimens per species; Ross and Mullins 1988); (3) additionally, *B. germanica* primarily inhabits indoor environments (Tang et al. 2019), while *B. asahinai* predominantly lives outdoors (Richard et al. 1988). In this study, specimens of *B. germanica* used for morphological observations and COI sequencing were laboratory-reared, while specimens of *B. asahinai* were collected from wild forest habitats in Yunnan Province. Given the limited length (658 bp) of the COI sequences and the nearly exclusive Chinese origin of the samples, we recommend expanding genetic sampling across broader geographical ranges and incorporating morphological analyses, supplemented by whole-genome sequencing approaches, to clarify the relationship between *B. germanica* and *B. asahinai*. Despite these limitations, the extremely low genetic distance (0.92%) between *B. germanica* and *B. asahinai*, coupled with their high morphological congruence, distinct ecological niches, and successful laboratory hybridization yielding viable F1 and F2 generations (Roth 1986), strongly support reclassifying *B. asahinai* as a subspecies of *B. germanica* (see Taxonomy section).

Based on the combined results of molecular analysis and morphological data, we successfully classified morphologically similar samples into 28 species (including one potential subspecies). The ABGD results corroborated the morphological delimitations, demonstrating that molecular data (COI) are effective for distinguishing *B. germanica* from its morphologically similar species. The intra- and interspecific genetic distances of 75 ingroup sequence are provided in Suppl. material 1. The interspecific genetic distances observed in this study ranged from 5.61% (between *B. nipponica* Asahina, 1963 and *B. bisignata* (Brunner von Wattenwyl, 1893) and between *E. paradoxura* Bey-Bienko, 1950 and *E. spinosa* (Bey-Bienko, 1969)) to 23.27% (between *B. radicifera* (Hanitsch, 1928) and *B. confusa* Princis, 1950). For *B. germanica*, the smallest genetic distance was 9.44% (to *B. bisignata*), while the largest was 20.81% (to *E. brevis* Qiao & Che, 2022).

## ﻿Taxonomy

### ﻿ Jacobsonina uncata

Taxon classificationAnimaliaBlattodeaBlattellidae

Cai, Yao & Che sp. nov.

FF93F04E-68B3-5F38-9C0C-871FBA1E5520

https://zoobank.org/6C27ABAD-966B-4DD9-83EB-12E1F878DD8E

Fig. 19A–J

#### Type material.

***Holotype***: China • male; Yunnan Province, Xishuangbanna City, Jinuo Township, Situ Xinzhai; 13 July 2020; Li-Kang Niu, Yi-Shu Wang leg. ***Paratypes***: China • 1 male & 1 female, same collection data as holotype.

#### Measurements

**(mm).** Male, overall length (including tegmen): 11.2, tegmina length: 8.8, pronotum length × width: 2.3 × 2.5.

#### Diagnosis.

The principal morphological distinction between *J. uncata* sp. nov. and congeneric species resides in the medial region of posterior margin of the supra-anal plate: *Jacobsonina* species generally exhibit either a slight concavity or rounded convexity, whereas *J. uncata* sp. nov. displays a pronounced concave configuration. This species resembles *Jacobsonina tortuosa* Wang, Jiang & Che, 2009 in body size and coloration, but can be distinguished by the following characteristics: 1) *J. uncata* sp. nov. with spines on the posterior lateral corner of the supra-anal plate (absent in *J. tortuosa*); 2) the hook phallomere of *J. uncata* sp. nov. with two hairy accessory sclerites, whereas only one accessory sclerite in *J. tortuosa*; 3) the conspicuous curved hook structure on the posterior margin of right paraproct in *J. uncata* sp. nov. (absent in *J. tortuosa*).

#### Description.

**Figure 19.**
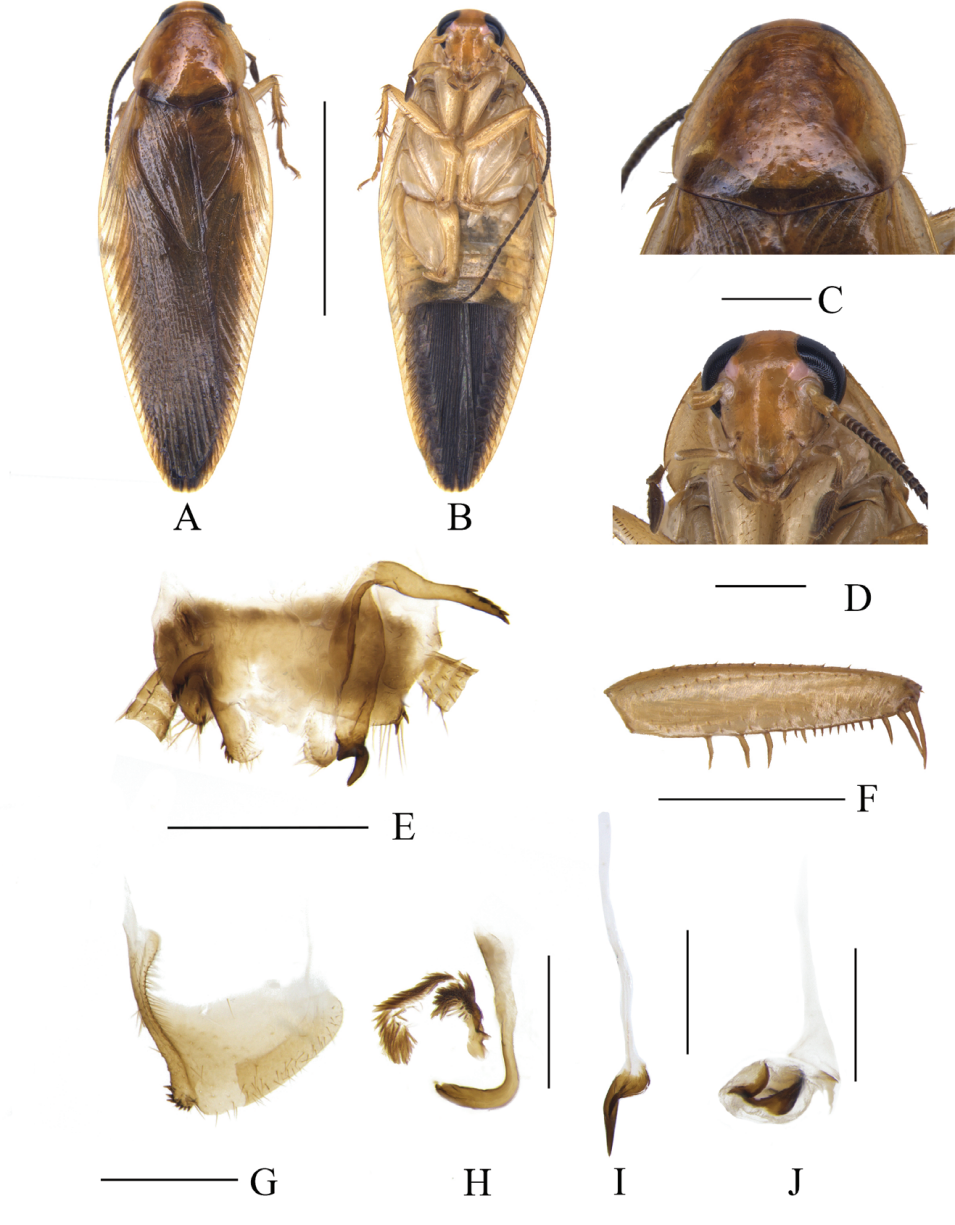
Holotype of *Jacobsonina uncata* Cai, Yao & Che, sp. nov. A. Habitus, dorsal view; B. Habitus, ventral view; C. Pronotum, dorsal view; D. Head, ventral view; E. Supra-anal plate, ventral view; F. Front femur, ventral view; G. Subgenital plate, ventral view; H. Left phallomere, ventral view; I. Median phallomere, ventral view; J. Right phallomere, ventral view. Scale bars: 5 mm (A, B); 1 mm (C–J).

**Male. *Coloration*.** Body yellowish. (Fig. 19A, B). Head and face yellowish brown. Pronotum yellowish brown, without distinct markings. Ocelli pink. Basal part of antennae yellowish brown. Maxillary palpi dark brown (Fig. 19C, D). Abdomen pale brown. Legs yellowish brown (Fig. 19F).

***Head*.** Interocular space about same as the distance between antennal sockets, slightly narrower than the distance between ocelli. ***Pronotum*.** Anterior margin slightly convex, lateral margins rounded, posterior margin slightly protruded medially (Fig. 19C). ***Tegmina and wings*.** Both fully developed, extending beyond the end of abdomen. ***Legs*.** Anteroventral margin of front femur Type B_3_ (Fig. 19F). Pulvilli present. Tarsal claws symmetrical and unspecialized, with arolia present. ***Abdomen and genitalia*.** Abdominal tergum unspecialized. Supra-anal plate unsymmetrical, left posterior lateral corner with two spines, the right with three spines. Paraprocts dissimilar. Left paraproct specialized, with apical part and distal part both hooked; the former comparatively short, strongly bent with the apex smooth and directed to the right; the latter strongly elongated and curved to the left, four spines present near the apex. Right paraproct simple, apical part with four stout spines; basal part with the hooked projection slightly curved, inner margin with four spines present (Fig. 19E). Subgenital plate unsymmetrical with left side thickened, left posterior lateral corner with several spines; styli absent (Fig. 19G). The terminal incision of the hook phallomere distinct, two accessory sclerites with long hairs (Fig. 19H). Median phallomere slender, the basal part widened at base and sharply narrowed at apex (Fig. 19I). Right phallomere complex: anterior sclerite weakly sclerotized and expanded basally; middle sclerite well sclerotized, posterior sclerite with small projection (Fig. 19J).

#### Etymology.

The specific epithet originates from the Latin term *uncatus*, which refers to the conspicuous curved hook structure on the posterior margin of right paraproct.

#### Distribution.

China (Yunnan).

### ﻿ Blattella subvittata

Taxon classificationAnimaliaBlattodeaBlattellidae

Hebard, 1929

2ABA7987-DCD2-5BCF-AE62-964DFF5C0CC4

Fig. 20A–O

Blattella subvittata Hebard, 1929: 58; Bruijning 1948: 36; Princis 1950: 176; Princis 1953: 55; Princis 1957: 144.
Blattella sauteri subvittata (Karny, 1915): Princis 1969: 844; Asahina 1981: 258.
Blattella subvittata Hebard: Roth 1985: 99.

#### Type locality.

“Pasoeroean, eastern Java”.

#### New material examined.

China • 1 male & 1 female; Hainan Province, Sanya City, Jiyang Town, Liupan Cun; 8 Apr. 2015; Xin-Ran Li, Zhi-Wei Qiu leg. • 1 female; Guangdong Province, Gaozhou City, Genzi Town; 11 June 2019; Rong Chen, Shan Gao leg. • 2 males; Hainan Province, Yinggeling, Nankai protection station; 14 July 2023; Yi-Shu Wang, Jin-Zhuo Cai leg. • 1 female; Hainan Province, Jianfeng Town; 18 July 2023; Yi-Shu Wang, Jin-Zhuo Cai leg.

**Figure 20.**
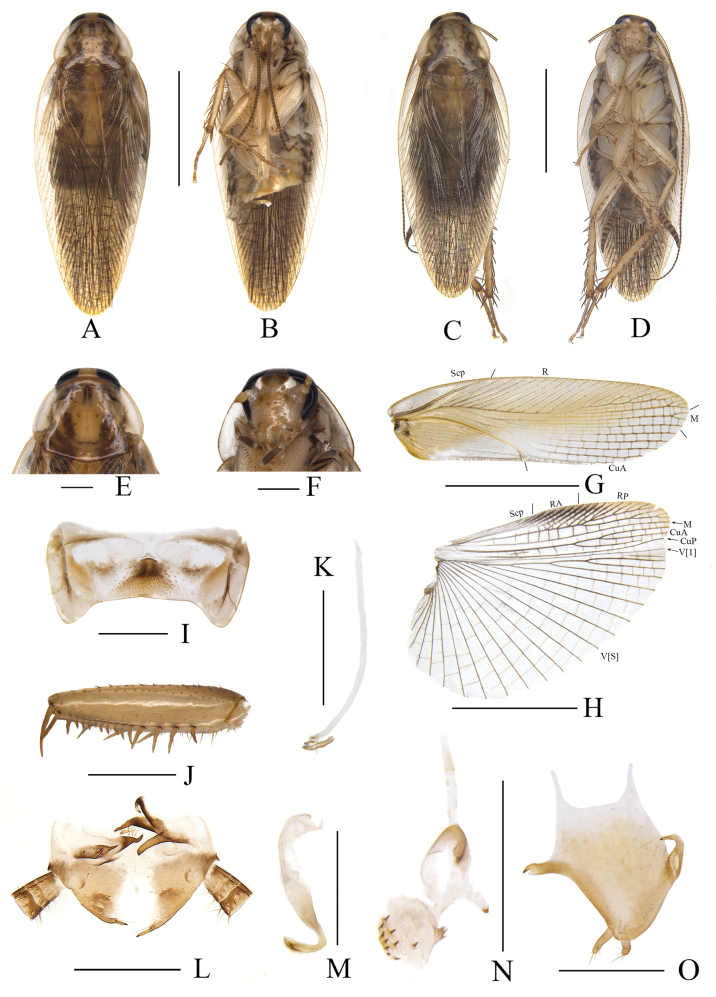
*Blattella subvittata* Hebard A, B, E–O. Male; C, D. Female. A. Habitus, dorsal view; B. Habitus, ventral view; C. Habitus, dorsal view; D. Habitus, ventral view; E. Pronotum, dorsal view; F. Head, ventral view; G. Tegmen, ventral view; H. Hind wing, ventral view; I. Abdominal tergite 7, ventral view; J. Front femur, ventral view; K. Median phallomere, ventral view; L. Supra-anal plate, ventral view; M. Left phallomere, ventral view; N. Right phallomere, ventral view; O. Subgenital plate, ventral view. Scale bars: 5 mm (A, B, C, D, G, H); 1 mm (E, F, I–O).

#### Distribution.

China (Guangdong, Hainan, Taiwan); Indonesia, Philippines, Malaysia, Thailand.

#### Remark.

Based on the markings of pronotum, the shape of abdominal tergite 7, and the structure of subgenital plate and supra-anal plate, samples from Hainan and Guangdong were identified as *B. subvittata*, with the closest genetic distance to *B. punctoria* (12.32%).

### ﻿ Blattella germanica asahinai

Taxon classificationAnimaliaBlattodeaBlattellidae

(Mizukubo, 1981) stat. nov.

0FFEADDB-1596-58BA-ACB7-8ADD0E0AAD93

Blattella asahinai Mizukubo, 1981: 153; Roth 2003: 87.
Blattella beybienkoi Roth, 1985: 28. Synonymized by Roth 1986: 371.

#### Material examined.

China • 3 females; Yunnan Province, Baoshan City, Baihualing, Jiu Jiezi; 24 Aug. 2015; Xin-Ran Li, Zhi-Wei Qiu leg. • 1 male; Yunnan Province, Dehong Dai and Jingpo Autonomous Prefecture, Yingjiang County, Nabang Town; 11 July 2012; Dong Wang leg.

#### Remarks.

Based on morphological comparisons, the genetic distance, and the ABGD delineation results, *B. asahinai* is considered to be a subspecies of *B. germanica*. The primary distinctions between *B. germanica* and *B. asahinai* are primarily observed in the morphology of the glandular fossae present on the eighth abdominal tergite, the varying sizes of their first ootheca, the coloration of the abdominal tergite during the later instars, and their distinct habitat environments (see Results and Fig. 18 for further details).

#### Distribution.

China (Taiwan, Yunnan); England, India, Indonesia, Japan, Malaysia, Myanmar, Thailand, United States.

## ﻿Conclusions

Our results indicate that the ABGD methodology generates species hypotheses for *B. germanica* and its morphologically similar species that are highly consistent with those derived from traditional morphological techniques. The DNA-based method demonstrates significant potential as a rapid, accurate, and autonomous identification tool for *B. germanica* and its morphologically similar species, making it particularly valuable for quarantine inspections at ports of entry. This study establishes a foundation for the identification of *B. germanica* and its morphologically similar species, thereby enhancing control and management efforts. Furthermore, our findings suggest that *B. asahinai* is not an independent species and should be classified as a subspecies of *B. germanica*. While this study advances the differentiation of *B. germanica* from its morphologically similar species, its scope is currently limited to those found in China. Broader sampling and further in-depth research, including the incorporation of additional molecular data for large-scale comparative analysis, are necessary to effectively distinguish these similar species.

## Supplementary Material

XML Treatment for Jacobsonina uncata

XML Treatment for Blattella subvittata

XML Treatment for Blattella germanica asahinai

## ﻿Additional information

### Use of AI

No use of AI was reported.

### Author ORCIDs

Jin-Zhuo Cai https://orcid.org/0009-0007-2343-9517

Wen-Wen Yao https://orcid.org/0009-0006-9329-8329

Zong-Qing Wang https://orcid.org/0000-0001-9413-1105

Yan-Li Che https://orcid.org/0000-0003-3214-9494

### Data availability

All of the data that support the findings of this study are available in the main text or Supplementary Information.

## Supplementary materials

Supplementary material 1 Genetic distances between COI gene of *B. germanica* and its morphologically similar relatives Data type xlsx https://binary.pensoft.net/file/1403088This dataset is made available under the Open Database License (http://opendatacommons.org/licenses/odbl/1.0/). The Open Database License (ODbL) is a license agreement intended to allow users to freely share, modify, and use this Dataset while maintaining this same freedom for others, provided that the original source and author(s) are credited.Jin-Zhuo Cai, Wen-Wen Yao, Zong-Qing Wang, Yan-Li Che

## References

[B1] Akinjogunla OJ Odeyemi AT Udoinyang EP (2012) Cockroaches (*Periplaneta americana* and *Blattella germanica*): Reservoirs of multidrug resistant (MDR) bacteria in Uyo, Akwa Ibom State. Scientific Journal of Biological Sciences 1(2): 19–30.

[B2] Asahina S (1981) Notes on the *Blattella* species of Taiwan. II. What is “*Ischnoptera sauteri* Karny 1915?”. Japanese Journal of Sanitary Zoology 32(4): 255–259. 10.7601/mez.32.255

[B3] Bai QK Wang LL Wang ZQ Lo N Che YL (2018) Exploring the diversity of Asian *Cryptocercus* (Blattodea: Cryptocercidae): species delimitation based on chromosome numbers, morphology and molecular analysis. Invertebrate Systematics 32(1): 69–91. 10.1071/IS17003

[B4] Bruijning CFA (1948) Studies on Malayan Blattidae-Zool. Rijksmuseum van Natuurlijke Historie, Leiden 29: 1–174.

[B5] Deng WB Liu YC Wang ZQ Che YL (2020) Eight new species of the genus *Anaplecta* Burmeister, 1838 (Blattodea: Blattoidea: Anaplectidae) from China based on molecular and morphological data. European Journal of Taxonomy 720: 77–106. 10.5852/ejt.2020.720.1117

[B6] Gore JC Schal C (2007) Cockroach allergen biology and mitigation in the indoor environment. Annual Review of Entomology 52(1): 439–463. 10.1146/annurev.ento.52.110405.091313 17163801

[B7] Han W Qiu L Zhu J Wang ZQ Che YL (2022) Exploring the diversity of *Eupolyphaga* Chopard, 1929 (Blattodea, Corydioidea): Species delimitation based on morphology and molecular analysis. ZooKeys 1120: 67–94. 10.3897/zookeys.1120.87483 36760327 PMC9848677

[B8] Han W Che YL Zhang PJ Wang ZQ (2024) New species of *Eupolyphaga* Chopard, 1929 and *Pseudoeupolyphaga* Qiu & Che, 2024 (Blattodea, Corydioidea, Corydiinae), with notes on their female genitalia. ZooKeys 1211: 151–191. 10.3897/zookeys.1211.128805 39268010 PMC11391126

[B9] Hebard M (1929) Studies in Malayan Blattidae (Orthoptera). Proceedings. Academy of Natural Sciences of Philadelphia 81: 1–109.

[B10] Karny H (1915) Sauter’s Formosa-Ausbeute. Orthoptera et Oothecaria. Supplementa Entomologica 4: 56–108.

[B11] Kimura M (1980) A simple method for estimating evolutionary rates of base substitutions through comparative studies of nucleotide sequences. Journal of Molecular Evolution 16(2): 111–120. 10.1007/BF01731581 7463489

[B12] Klass KD (1997) The external male genitalia and the phylogeny of Blattaria and Mantodea. Bonner Zoologische Monographien 42: 1–341.

[B13] Kumar NP Rajavel AR Natarajan R Jambulingam P (2007) DNA barcodes can distinguish species of Indian mosquitoes (Diptera: Culicidae). Journal of Medical Entomology 44(1): 1–7. 10.1093/jmedent/41.5.01 17294914

[B14] Lanfear R Frandsen PB Wright AM Senfeld T Calcott B (2017) PartitionFinder 2: New methods for selecting partitioned models of evolution for molecular and morphological phylogenetic analyses. Molecular Biology and Evolution 34(3): 772–773. 10.1093/molbev/msw260 28013191

[B15] Li XR Zheng YH Wang CC Wang ZQ (2018) Old method not old-fashioned: Parallelism between wing venation and wing-pad tracheation of cockroaches and a revision of terminology. Zoomorphology 137(4): 519–533. 10.1007/s00435-018-0419-6

[B16] Li QQ Yao WW Zhang K Wang ZQ Che YL (2023) Six new species of *Margattea* Shelford, 1911 (Blaberoidea, Pseudophyllodromiidae, Neoblattellini) from China. ZooKeys 1191: 339–367. 10.3897/zookeys.1191.113147 PMC10892154 38405678

[B17] McKittrick FA (1964) Evolutionary studies of cockroaches. Memoirs of the Cornell University Agricultural Experiment Station 389: 1–197.

[B18] Mizukubo T (1981) A revision of the genus *Blattella* (Blattaria: Blattellidae) of Japan, I. terminology of the male genitalia and description of a new species from Okinawa Island. Kyushu University Institutional Repository 17: 149–159. 10.5109/2418

[B19] Moges F Eshetie S Endris M Huruy K Muluye D Feleke TG Silassie F Ayalew G Nagappan R (2016) Cockroaches as a source of high bacterial pathogens with multidrug resistant strains in Gondar town, Ethiopia. BioMed Research International 2016: 1–6. 10.1155/2016/2825056 PMC4909895 27340653

[B20] Naqqash MN Saeed Q Saeed S Jaleel W Zaka SM Faheem M Bakhtawar M Rehman S (2014) A cross sectional survey of community awareness about typhoid and its major vector cockroach in southern punjab, Pakistan. Middle East Journal of Scientific Research 21(4): 602–608.

[B21] Nguyen LT Schmidt HA Von Haeseler A Minh BQ (2014) IQ-TREE: A fast and effective stochastic algorithm for estimating maximum-likelihood phylogenies. Molecular Biology and Evolution 32(1): 268–274. 10.1093/molbev/msu300 25371430 PMC4271533

[B22] Princis K (1950) Indomalaische und australische Blattarienaus dem Entomologischen Museum der Lund. Opuscula Entomologica 15: 161–188.

[B23] Princis K (1953) Kleine Beitröge zur Kenntnis der Blottorien und ihrer Verbreitung. Opuscula Entomologica 18: 53–58.

[B24] Princis K (1957) Zur Kenntniss der Blattarien der Kleinen Sundainseln. Verhandlungen der Naturforschenden Gesellschaft in Basel 68: 132–159.

[B25] Princis K (1969) Blattariae: Subordo Epilamproidea, Fam.: Blattellidae. Orthopterorum Catalogus 13: 712–1038.

[B26] Puillandre N Lambert A Brouillet S Achaz G (2012) ABGD, automatic barcode gap discovery for primary species delimitation. Molecular Ecology 21(8): 1864–1877. 10.1111/j.1365-294X.2011.05239.x 21883587

[B27] Ren CH Chen N Li S (2023) Harnessing “little mighty” cockroaches: Pest management and beneficial utilization. Innovation 4(6): 1–2. 10.1016/j.xinn.2023.100531 PMC10661541 38028134

[B28] Richard J Brenner RS Patterson PG (1988) Koehler, ecology, behavior, and distribution of *Blattella asahinai* (Orthoptera: Blattellidae) in central Florida. Annals of the Entomological Society of America 3(81): 432–436. 10.1093/aesa/81.3.432

[B29] Ross MH Mullins DE (1988) Nymphal and oöthecal comparisons of *Blattella asahinai* and *Blattella germanica* (Dictyoptera: Blattellidae). Journal of Economic Entomology 81(6): 1645–1647. 10.1093/jee/81.6.1645

[B30] Roth LM (1985) A taxonomic revision of the genus *Blattella* Caudell (Dictyoptera: Blattaria: Blattellidae). Atherosclerosis. Supplements 22: 1–221.

[B31] Roth LM (1986) *Blattella asahinai* introduced into Florida (Blattaria: Blattellidae). Psyche 93(3–4): 371–374. 10.1155/1986/60130

[B32] Roth LM (2003) Systematics and phylogeny of cockroaches (Dictyoptera: Blattaria). Oriental Insects 37(1): 1–186. 10.1080/00305316.2003.10417344

[B33] Santos ABR Chapman MD Aalberse RC Vailes LD Ferriani VP Oliver C Rizzo MC Naspitz CK Arruda LK (1999) Cockroach allergens and asthma in Brazil: Identification of tropomyosin as a major allergen with potential cross-reactivity with mite and shrimp allergens. The Journal of Allergy and Clinical Immunology 104(2): 329–337. 10.1016/S0091-6749(99)70375-1 10452753

[B34] Tang Q Bourguignon T Willenmse L De Coninck E Evans TA (2019) Global spread of the German cockroach, *Blattella germanica*. Biological Invasions 21(3): 693–707. 10.1007/s10530-018-1865-2

[B35] Tang Q Vargo EL Ahmad I Jiang H Varadínová ZK Dovih P Kim D Bourguignon T Booth W Schal C Mukha DV Rheindt FE Evans TA (2024) Solving the 250-year-old mystery of the origin and global spread of the German cockroach, *Blattella germanica*. Proceedings of the National Academy of Sciences of the United States of America 121(22): 1–3. 10.1073/pnas.2401185121 PMC11145273 38768340

[B36] Wang ZQ Jiang HY Che YL (2009) Two new species and one new record of the genus *Jacobsonina* Hebard (Blattaria, Blattellidea) from China. Dong Wu Fen Lei Xue Bao 34(4): 751–756.

[B37] Wang ZQ Che YL Feng PZ (2010) The taxonomic study of the genus *Blattella* Caudell 1903 from China with description of one new species (Blattaria: Blattellidae). Acta Entomologica Sinica 53(8): 908–913.

[B38] Wang YS Chen R Jin DT Che YL Wang ZQ (2021) New record of *Cyrtonotula* Uvarov, 1939 (Blaberidae, Epilamprinae) from China, with three new species based on morphological and COI data. ZooKeys 1021: 127–143. 10.3897/zookeys.1021.59526 33727886 PMC7943533

[B39] Wang YS Zhang JW Lo N Bourguignon T Guo L Li BL Che Y-L Wang Z-Q (2023) Phylogenetic analysis of Blaberoidea reveals non‐monophyly of taxa and supports the creation of multiple new subfamilies. Cladistics 39(3): 198–214. 10.1111/cla.12535 37067219

[B40] Yao WW Li QQ Li LW Wang ZQ Che YL (2024) Discovery of four new Chinese species of *Jacobsonina* Hebard, 1929 and *Blattella* Caudell, 1903 (Blaberoidea: Blattellidae). Zootaxa 5474(4): 427–440. 10.11646/zootaxa.5474.4.5 39646481

[B41] Zhang D Gao F Jakovlic I Zo H Zhang J Li WX Wang GT (2020) PhyloSuite: An integrated and scalable desktop platform for streamlined molecular sequence data management and evolutionary phylogenetics studies. Molecular Ecology Resources 20(1): 348–355. 10.1111/1755-0998.13096 31599058

[B42] Zhu J Zhang JW Luo XX Wang ZQ Che YL (2022) Three cryptic *Anaplecta* (Blattodea, Blattoidea, Anaplectidae) species revealed by female genitalia, plus seven new species from China. ZooKeys 1080: 53–97. 10.3897/zookeys.1080.74286 35068964 PMC8752576

